# An Experimental and Master Equation Investigation of Kinetics of the CH_2_OO + RCN Reactions (R = H, CH_3_, C_2_H_5_) and Their Atmospheric Relevance

**DOI:** 10.1021/acs.jpca.2c07073

**Published:** 2023-01-05

**Authors:** Lauri Franzon, Jari Peltola, Rashid Valiev, Niko Vuorio, Theo Kurtén, Arkke Eskola

**Affiliations:** Department of Chemistry, University of Helsinki, P.O. Box 55 (A.I. Virtasen aukio 1), 00014 Helsinki, Finland

## Abstract

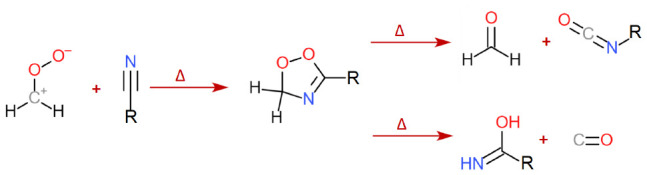

We have performed direct kinetic measurements of the CH_2_OO + RCN reactions (R = H, CH_3_, C_2_H_5_) in the temperature range 233–360 K and pressure range 10–250 Torr using time-resolved UV-absorption spectroscopy. We have utilized a new photolytic precursor, chloroiodomethane (CH_2_ICl), whose photolysis at 193 nm in the presence of O_2_ produces CH_2_OO. Observed bimolecular rate coefficients for CH_2_OO + HCN, CH_2_OO + CH_3_CN, and CH_2_OO + C_2_H_5_CN reactions at 296 K are (2.22 ± 0.65) × 10^–14^ cm^3^ molecule^–1^ s^–1^, (1.02 ± 0.10) × 10^–14^ cm^3^ molecule^–1^ s^–1^, and (2.55 ± 0.13) × 10^–14^ cm^3^ molecule^–1^ s^–1^, respectively, suggesting that reaction with CH_2_OO is a potential atmospheric degradation pathway for nitriles. All the reactions have negligible temperature and pressure dependence in the studied regions. Quantum chemical calculations (ωB97X-D/aug-cc-pVTZ optimization with CCSD(T)-F12a/VDZ-F12 electronic energy correction) of the CH_2_OO + RCN reactions indicate that the barrierless lowest-energy reaction path leads to a ring closure, resulting in the formation of a 1,2,4-dioxazole compound. Master equation modeling results suggest that following the ring closure, chemical activation in the case of CH_2_OO + HCN and CH_2_OO + CH_3_CN reactions leads to a rapid decomposition of 1,2,4-dioxazole into a CH_2_O + RNCO pair, or by a rearrangement, into a formyl amide (RC(O)NHC(O)H), followed by decomposition into CO and an imidic acid (RC(NH)OH). The 1,2,4-dioxazole, the CH_2_O + RNCO pair, and the CO + RC(NH)OH pair are atmospherically significant end products to varying degrees.

## Introduction

The ozonolysis of alkenes, i.e., O_3_ + alkene → products, is a central reaction in atmospheric chemistry. Ozonolysis leads to the formation of highly reactive Criegee Intermediates (CI; carbonyl oxides with the general structure R_1_R_2_COO), which can undergo a large variety of subsequent reactions.^1^ The atmospheric fate of especially larger CIs is often chemically and thermally activated isomerization followed by a rapid unimolecular decomposition.^2^ A significant fraction of CIs can be thermalized by collisions with inert gas molecules, leading to stabilized Criegee Intermediates (sCIs). The smallest CI, CH_2_OO, formed in the ozonolysis of all terminal alkenes,^3^ has a substantial sCI yield due to the lack of low-energy barrier channel for isomerization or decomposition. Bimolecular reactions of sCIs are of great interest,^4^ as their unusual electronic structure enables oxidation mechanisms which would otherwise not occur in atmospheric conditions. In this article, the bimolecular reaction of CH_2_OO with hydrogen cyanide, acetonitrile, and propionitrile (HCN, CH_3_CN, and C_2_H_5_CN) is studied, from both experimental and computational perspectives.

HCN and CH_3_CN are released into the troposphere mainly from biomass burning and to a lesser extent from other sources. Both species are chemically quite stable in atmospheric conditions and, consequently, are used as reliable tracers of biomass burning emissions.^5−7^ Nitriles are also a well-known industrial reagent in rubber production^8^ and this is a potential atmospheric source as well, but field experiments in heavily populated areas^9^ indicate that this source is insignificant compared to biomass burning. Another source of nitriles in general and HCN in particular is the OH-radical initiated oxidation of imines,^10,11^ which are one of the primary oxidation products of amines in the atmosphere.^12^ Atmospheric degradation of amines is currently a subject of considerable interest, because significant amounts of amines are expected to be released into the atmosphere from utilization of postcombustion Carbon Capture technology. These approaches aim to separate atmosphere-heating CO_2_ from flue gases of large-scale combustion facilities before subsequent treatment of the highly enriched CO_2_.^13^

Atmospheric reaction chemistry of nitriles has received attention before.^14,15^ HCN has a relatively long lifetime in the stratosphere, limited by a rapid reaction with O(^1^D), but in the troposphere the main sink is the ocean uptake rather than gas-phase reactions, resulting in a lifetime of a few months.^16^ A significant ocean uptake is hypothesized for acetonitrile as well.^17^ The most significant gas-phase reaction of HCN in the troposphere is with the OH radical, *k*_r_ = 8.98 × 10^–15^ cm^3^ molecule^–1^ s^–1^ at the high-pressure limit and *T* = 298 K.^15^ Reactions with NO_*x*_ have been hypothesized, but combustion experiments have shown that these reactions have very high activation energies.^18,19^ The atmospheric chemistry of nitriles with longer alkyl substituents is less investigated, but some experimental data exist on the most important property for our purposes, i.e., for the bimolecular rate coefficient with the OH and Cl radicals.^14^ The room temperature rate coefficient of OH + RCN reactions seems to increase by approximately a factor of 4 for every −CH_2_– unit added to the alkyl group: *k*_r_(OH + CH_3_CN) = 4 × 10^–14^ cm^3^ molecule^–1^ s^–1^, *k*_r_(OH + C_2_H_5_CN) = 1.27 × 10^–13^ cm^3^ molecule^–1^ s^–1^, while the rate coefficient of Cl + RCN reactions increases by an order of magnitude for every −CH_2_– added: starting from *k*_r_(Cl + CH_3_CN) = 1.1 × 10^–14^ cm^3^ molecule^–1^ s^–1^) and the fastest measured being *k*_r_(Cl + C_4_H_9_CN) = 6.7 × 10^–11^ cm^3^ molecule^–1^ s^–1^. The Cl radical is much less abundant in the atmosphere than the OH radical, so higher rate coefficients do not immediately imply higher degradation rates, but these results may suggest that the CH_2_OO + RCN reaction is potentially less likely to be atmospherically significant for nitriles with longer alkyl substituents. This was the main reason for limiting the current study to the three smallest nitrile compounds. A very recent theoretical study by Zhang et al.^20^ has investigated the reactions of CH_2_OO and acetaldehyde oxide (CH_3_CHOO) with acetonitrile. Their value for the high-pressure-limit rate coefficient of CH_2_OO + CH_3_CN reaction at 298 K is 1.16 × 10^–14^ cm^3^ molecule^–1^ s^–1^. They also state that the reaction possesses a weak negative temperature dependence.

In addition to the Zhang et al.^20^ study on the CH_2_OO + CH_3_CN reaction, Sun et al.^21^ have also investigated theoretically the bimolecular reaction of CH_2_OO with a triple bond, the reaction CH_2_OO + C_2_H_2_. In both cases, the reaction proceeds to the formation of a five-membered ring. To our knowledge, the current work is the first direct experimental study of sCI reaction with triple bond compounds.
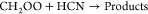
R1
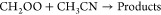
R2
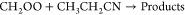
R3

## Experimental Methods

**Figure 1:**
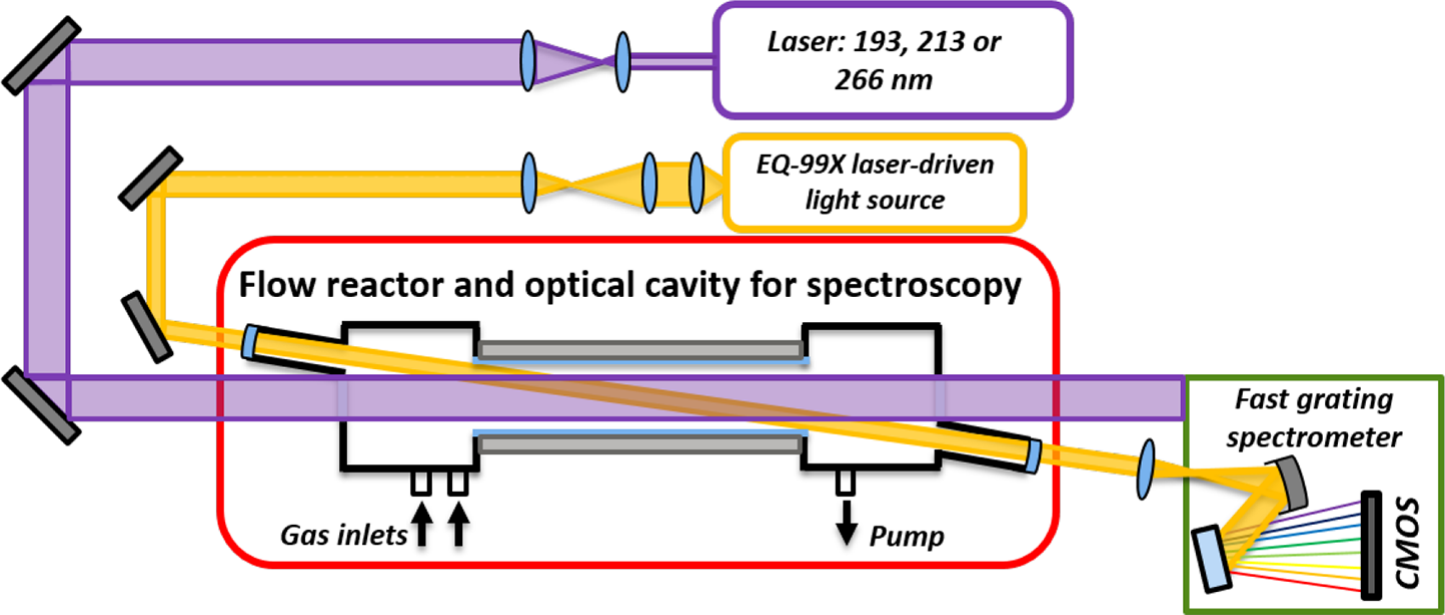
Schematic figure of the time-resolved broadband cavity-enhanced absorption spectrometer. The sCI, CH_2_OO, is produced along a heated or cooled flow tube reactor by a single-pass photolysis laser pulse at 193, 213, or 266 nm. The sCI is probed by overlapping incoherent laser-driven broadband light source. The sensitivity of the detection is enhanced using an optical cavity formed by two concave mirrors, highly reflecting between 300 and 450 nm. The time-dependent broadband absorption spectrum of sCI is measured by a grating spectrometer combined with a fast CMOS line array camera. Reproduced with permission from ref (22). Copyright 2020 The Royal Society of Chemistry.

**Figure 2:**
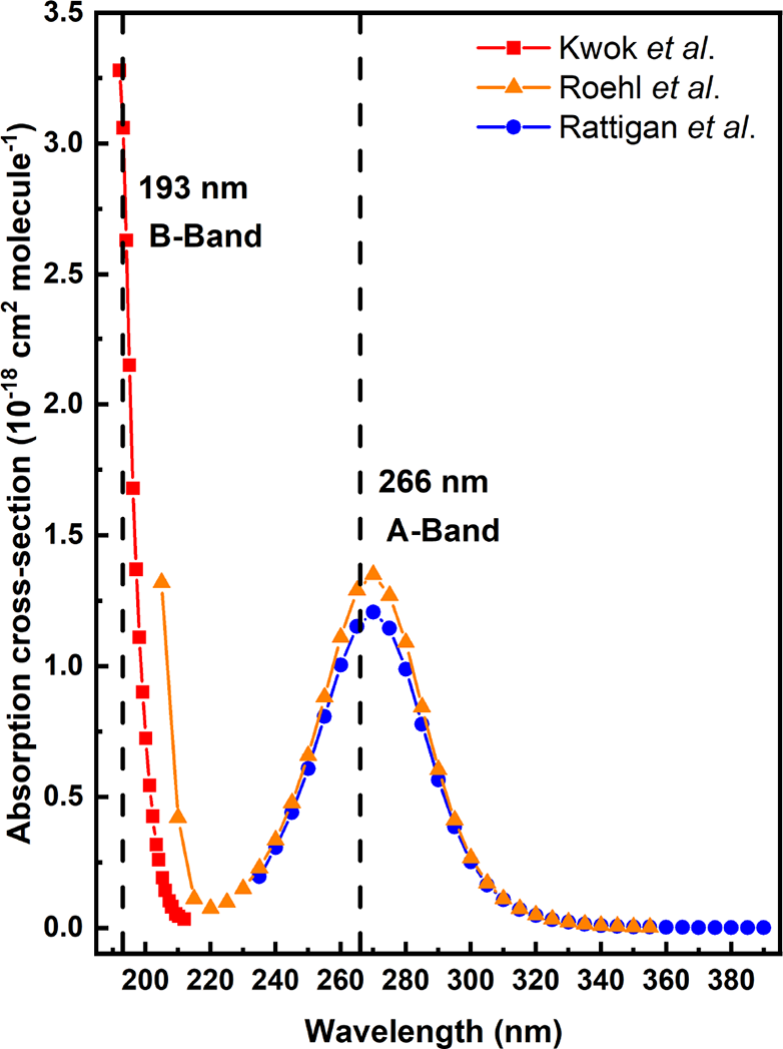
UV absorption cross-section of gaseous CH_2_ICl as a function of wavelength measured by Kwok et al.^27^ (red squares), Roehl et al.^28^ (orange triangles), and Rattigan et al.^29^ (blue circles).

The kinetics of reactions R1–R3 were measured using a time-resolved, broadband, cavity-enhanced absorption spectrometer (TR-BB-CEAS) that is schematically shown in Figure 1 and has been described previously.^22,23^ The absorption of the smallest sCI, formaldehyde oxide, CH_2_OO, was followed using TR-BB-CEAS. CH_2_OO was produced in a fast two-step process; first generating CH_2_I radical photolytically from a precursor, followed by rapid reaction of CH_2_I radical with O_2_ to produce CH_2_OO.^24^
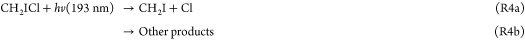
R4afollowed by
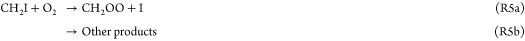
R5aChloroiodomethane (CH_2_ICl, purity , TCI) was the main photolytic precursor of CH_2_OO in this work. Eskola et al.^25^ have found that the photodissociation of CH_2_ICl at 193 nm (B-band, see Figure 2) also produces CH_2_Cl, CHCl, and CH_2_, but their concentrations are small and did not have any significant effect on the current measurements (see more in Experimental Results). It is also known that Cl atoms react rapidly with CH_2_ICl to produce CH_2_Cl and ICl.^26^ The UV absorption cross-section of gaseous CH_2_ICl as a function of wavelength is presented in Figure 2. As with the CH_2_IBr precursor used in our previous studies, the absorption cross-section of CH_2_ICl at 340 nm region (the absorption maximum of CH_2_OO) is significantly smaller than the cross-section of CH_2_I_2_ (see Figure S1 in the Supporting Information), resulting in a zero or small positive and constant baseline for the measured CH_2_OO absorption signal, while CH_2_I_2_ results in a negative (and non-constant) baseline.^22^

A known dilution (typically ∼1 Torr/1000 Torr) of the photolytic precursor (in helium) was prepared in a 3.5-L glass container and the mixture was flowed through the reactor being diluted further with nitrogen buffer gas. The CH_2_ICl precursor was photolyzed by an ArF exciplex laser (MPB Technologies ASX-750) at 193 nm in the presence of a large excess of O_2_ ([O_2_] ≫ [CH_2_I]). The laser fluences used were between 6 and 25 mJ cm^–2^. In a few measurements, CH_2_IBr and CH_2_I_2_ precursors were used for comparison. In these measurements, the CH_2_I radicals were generated by the fifth harmonic (213 nm) or the fourth harmonic (266 nm) of a pulsed Nd:YAG laser (Quantel Q-smart 850). The HCN reagent was introduced from a gas cylinder (standard mixture of 350 ppm), the concentration of which was verified in a separate FTIR experiment. The CH_3_CN or CH_3_CH_2_CN nitrile reagent was supplied to the reactor by bubbling nitrogen gas at known pressure and flow rate through a temperature-stabilized liquid nitrile reagent with known vapor pressure at the used temperature. The gas flows were controlled with calibrated mass-flow controllers, and the total gas-mixture was preheated or precooled close to a set point temperature before entering into the temperature-controlled reactor. The total flow of about 1 ms^–1^ was used to replace the gas-mixture between laser pulses with a repetition rate of 1 Hz. All the kinetic traces of CH_2_OO were measured at 338 nm. For the experiments described here, we averaged signal between 1000 and 6000 shots for each decaying experimental time-trace. The time-traces were probed with a time resolution of 160 μs.

## Computational Methods

Since the current experimental setup was used to measure kinetics of reactions R1–R3 by following time-behavior of [CH_2_OO], quantum chemical calculations and master equation (ME) simulations were also performed to determine the reaction mechanism and products over a *p*, *T*-range relevant for tropospheric and stratospheric conditions. The ME simulations were performed with the MESMER 6.0 program, which is a one-dimensional ME code.^30^

Due to the challenging electronic structure of some important transition states, three different levels of quantum chemical theory were used to calculate potential energy surfaces for the reactions R1–R3: density functional theory (DFT; specifically ωB97X-D/aug-cc-pVTZ) with coupled-cluster (CCSD(T)-F12a/VDZ-F12) energy corrections, extended multiconfiguration quasi-degenerate perturbation theory (XMC-QDPT2), and the spin-flip approach in time-dependent density functional theory (SF-TDDFT) at the B3LYP/aug-cc-pVTZ level of theory. The first of these was implemented on all reactants, intermediates, products, and transition states. The latter two methods were only used for the intermediates and transition states whose energies had a crucial impact on the identity of final products in atmospheric conditions. See the Supporting Information for specific details on each set of calculations.

The ME simulations of the CH_2_OO + RCN reactions were performed using the energies calculated with all three methods, CCSD(T), XMC-QDPT2, and SF-TDDFT. The rigid rotor harmonic oscillator approximation was utilized using the vibrational frequencies and rotational constants from the quantum chemical geometry optimizations. For each species in the ME, the most accurate set of vibrational frequencies available was used. These were the XMC-QDPT2 frequencies for the systems on which these calculations were performed (i.e., for the 1,2,4-dioxazole and its two decomposition transition states. See our section on Quantum Chemical Results for a full discussion of the reaction mechanism) and the ωB97X-D/aug-cc-pVTZ level frequencies for all other systems. Collisional energy transfer between reaction intermediates and bath gas molecules was modeled using the exponential-down model with Lennard-Jones collision frequencies.^31^ The average energy transferred in a collision, ⟨Δ*E*⟩_down_, was estimated for the relevant intermediates based on the amount of non-H atoms and on the presence or absence on ring structures, using Jasper et al.’s extensive dataset on energy transfer parameters of hydrocarbons in different bath gases.^32^ The exact values used in this work are tabulated in Table S11; in short, ⟨Δ*E*⟩_down_ values in the range 260–340 cm^–1^ were used for cyclic structures and 490–570 cm^–1^ for acyclic structures. A sensitivity analysis was performed on the effect of ⟨Δ*E*⟩_down_ values on the product distribution. The results are found in Table S12. The concentration of the excess reactant (the nitrile) was set at 10^15^ molecules cm^–3^ for all ME simulations. The Lennard-Jones parameters used for the intermediates are found in Table S10, and the method for calculating these is described in the Supporting Information.

The appropriate approach for modeling the initial barrierless association and the following reaction over a submerged barrier depends on the reaction energetics and on the thermodynamic conditions, especially on temperature. Georgievskii’s and Klippenstein’s Long-Range Transition State Theory^33^ derives the existence of both an outer transition state owing to centrifugal acceleration and an inner transition state owing to the chemical interaction of the two molecules. A previous study on a closely similar reaction system, the reaction between CH_2_OO and simple carbonyls,^34^ is referred here for determining the parameters for the initial association. A constant capture rate of  cm^3^ molecule^–1^ s^–1^ was used based on the results of Elsamra et al.^34^ The basis for assuming this value is discussed further in the Supporting Information.

Additional ME simulations were made in which the initial barrierless association and the following reaction over the submerged barrier to form a cyclic intermediate were modeled using the Inverse Laplace Transform (ILT) method.^35^ The parameters *A* and *n* in eq 1 were determined by least-squares fitting to the experimental kinetic data presented in Tables 2, 3, and 4 (*T*_ref_ = 298.15 K), and the resulting expression was transformed to microcanonical rates. Calculations with the ILT method were made for comparison with the method above. The comparison is presented in Tables S13 and S14 in the Supporting Information.
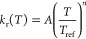
1

**Table 1:** Pseudo-first-order Decay Rate Coefficients (*k*_obs_) and Conditions Used to Measure Kinetics of the Bimolecular Reaction CH_2_OO+HCN. Concentrations Are Presented in molecule cm^–3^^a^

*T*(K)	[N_2_]/10^18^	*p*(Torr)	[HCN]/10^15^	*k*_obs_(s^–1^))
296	8.2	250	0	43 ± 3
296	8.2	250	0.70	70 ± 5
296	8.2	250	1.40	84 ± 7
296	8.2	250	2.80	108 ± 11

a CH_2_ICl precursor concentration was ∼ 2.0 × 10^12^ molecule cm^–3^. Estimated initial CH_2_OO concentration < 1.0 × 10^11^ molecule cm^–3^. The fixed O_2_ concentration was ∼ 4.0 × 10^16^ molecule cm^–3^.

**Table 2:** Obtained Bimolecular Rate Coefficients, *k*_r_ (cm^3^ molecule^–1^ s^–1^), and the Conditions Used for the Reaction of CH_2_OO + CH_3_CN as a Function of Temperature at Constant Density Utilizing Different Photolytic Precursors. Concentrations Are Presented in Molecule cm^–3^^a^

*T*(K)	[N_2_]/10^17^	*p*(Torr)	[CH_3_CN]/10^15^	*k*_loss_(s^–1^)	*k*_r_/10^–14^
Precursor: CH_2_ICl					
233	3.3	7.9	1.98–8.03	39 ± 2	1.28 ± 0.11
253	3.3	8.5	1.97–5.94	25 ± 8	1.29 ± 0.16
273	3.3	9.2	2.09–8.34	43 ± 2	1.04 ± 0.11
296	3.3	10	2.14–8.64	39 ± 2	0.92 ± 0.10
296	3.3	10	2.22–8.91	46 ± 3	1.12 ± 0.11
320	3.3	10.8	2.22–8.67	45 ± 3	1.47 ± 0.16
320	3.3	10.8	2.18–6.35	45 ± 3	1.21 ± 0.12
360	3.3	12.1	2.05–9.01	48 ± 3	1.11 ± 0.24
Precursor: CH_2_IBr					
296	3.3	10	3.03–15.3	62 ± 6	1.35 ± 0.12
353	3.3	11.9	3.03–15.2	49 ± 7	1.37 ± 0.12
Precursor: CH_2_I_2_					
296	3.3	10	3.07–15.5	36 ± 12	1.42 ± 0.10
353	3.3	11.9	3.17–15.9	46 ± 11	1.42 ± 0.20

a Precursor concentrations used: <1.0 × 10^12^ molecule cm^–3^ for CH_2_ICl, ∼3.0 × 10^13^ molecule cm^–3^ for CH_2_IBr and ∼8.0 × 10^12^ molecule cm^–3^ for CH_2_I_2_. Estimated initial CH_2_OO concentration <1.0 × 10^11^ molecule cm^–3^ when using CH_2_ICl precursor and <6.0 × 10^11^ molecule cm^–3^ when using CH_2_IBr and CH_2_I_2_ precursors. The fixed O_2_ concentration was ∼4.0 × 10^16^ molecule cm^–3^.

**Table 3:** Obtained Bimolecular Rate Coefficients, *k*_r_ (cm^3^ molecule^–1^ s^–1^), and the Conditions Used for the Reaction of CH_2_OO + CH_3_CH_2_CN as a Function of Temperature at Constant Density Utilizing Different Photolytic Precursors. Concentrations Are Presented in Molecule cm^–3^^a^

*T*(K)	[N_2_]/10^17^	*p*(Torr)	[CH_3_CH_2_CN]/10^15^	*k*_loss_(s^–1^)	*k*_r_/10^–14^
Precursor: CH_2_ICl					
233	3.3	7.9	0.99–3.01	42 ± 3	3.30 ± 0.46
253	3.3	8.5	1.05–4.22	45 ± 3	3.53 ± 0.26
273	3.3	9.2	1.04–4.23	40 ± 2	2.56 ± 0.12
296	3.3	10	1.02–4.41	39 ± 3	2.55 ± 0.13
320	3.3	10.8	1.08–4.34	45 ± 3	2.76 ± 0.44
360	3.3	12.1	1.08–4.27	40 ± 2	2.21 ± 0.11
Precursor: CH_2_IBr					
296	3.3	10	1.44–7.30	57 ± 6	3.16 ± 0.48
353	3.3	11.9	1.46–7.34	74 ± 4	2.48 ± 0.30
Precursor: CH_2_I_2_					
296	3.3	10	1.41–7.10	32 ± 10	2.41 ± 0.15
353	3.3	11.9	1.41–7.00	35 ± 9	3.06 ± 0.48

a Precursor concentrations used: <1.0 × 10^12^ molecule cm^–3^ for CH_2_ICl, ∼3.0 × 10^13^ molecule cm^–3^ for CH_2_IBr and ∼8.0 × 10^12^ molecule cm^–3^ for CH_2_I_2_. Estimated initial CH_2_OO concentration <1.0 × 10^11^ molecule cm^–3^ when using CH_2_ICl precursor and <6.0 × 10^11^ molecule cm^–3^ when using CH_2_IBr and CH_2_I_2_ precursors. The fixed O_2_ concentration was ∼4.0 × 10^16^ molecule cm^–3^.

## Results and Discussion

### Experimental Results

**Figure 3:**
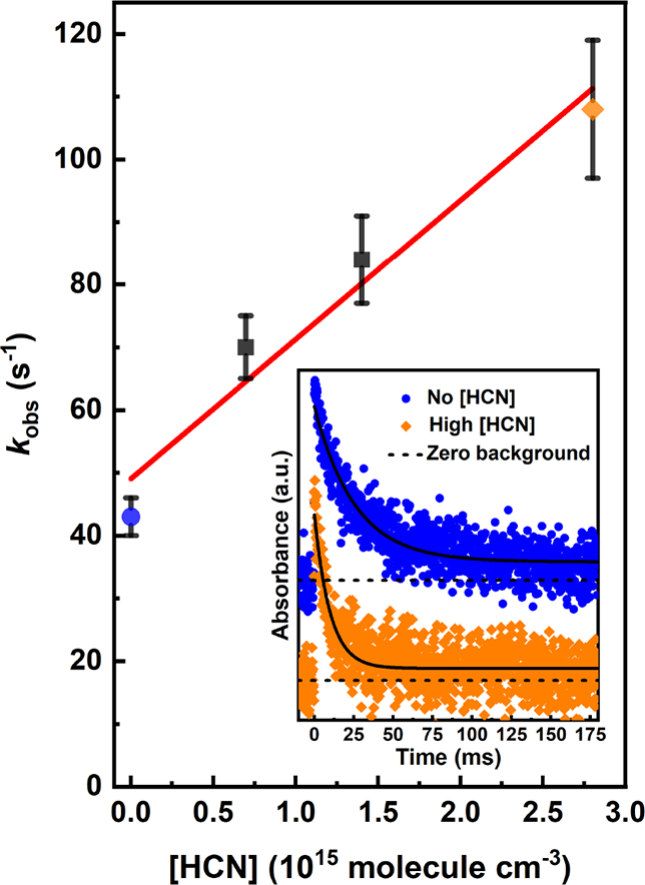
Determination of the bimolecular rate coefficient of CH_2_OO + HCN reaction from the plot of pseudo-first-order decay rate coefficients (*k*_obs_) versus [HCN] at 296 K and 250 Torr utilizing CH_2_ICl photolytic precursor. The [CH_2_OO] trace in the absence (blue) and presence (orange) of HCN is shown in the bottom right corner. The colored symbols (blue circle and orange diamond) in the figure depict the measurements that correspond to the shown traces. The traces are shifted vertically for clarity.

The kinetics of CH_2_OO reaction with CH_3_CN and CH_3_CH_2_CN were measured as a function of temperature between 233 and 360 K at low pressures (7.9–12.1 Torr) and keeping [total] approximately constant. The kinetics of CH_2_OO + HCN reaction was measured only at room temperature (296 K) and 250 Torr, because there was only enough gas in the HCN/N_2_ cylinder (350 ppm, 10 L) for one set of measurements. The bottom right corner of Figure 3 shows transient traces of CH_2_OO in the absence and presence of HCN. All the CH_2_OO traces in this study were fitted using single-exponential decay function
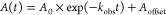
2where *k*_obs_ is the first-order decay rate coefficient to be obtained, *A*(*t*) is the absorbance at time *t*, *A*_0_ is the initial absorbance (at time *t* = 0), and *A*_offset_ is the constant absorbance caused by nonreactive species (formed at time *t* = 0). In the absence of added nitrile reactant, the CH_2_OO signal follows a first-order decay loss, *k*_loss_ (*s*^–1^), which was always measured at the beginning of each rate coefficient measurement. The *k*_loss_ includes the diffusion out of the measurement volume, a contribution from self-reaction of CH_2_OO, and the possible slow reaction of CH_2_OO with the precursor. To minimize the effect of self-reaction and Criegee-precursor reactions, low Criegee  and precursor  concentrations were typically used in the measurements. We also performed some experiments with higher precursor concentration, but with lower laser fluence, to test the importance of the Criegee-precursor CH_2_OO + CH_2_ICl reaction. The measured decay rate coefficients in the absence of nitrile reagent are shown in Table S1 as a function of [CH_2_ICl] at 296 K and 10 Torr. The measured decay rate coefficients do not depend on the [CH_2_ICl] to any significant extent. In addition, heterogeneous loss is negligible in our measurement system, since the radicals are generated and probed inside the same volume element in the middle of the flow reactor tube away from the walls. The unimolecular decay of CH_2_OO is also insignificant within the temperature range of this study.^22,36^

By adding HCN reagent, the decay of CH_2_OO became faster. All the measurements in this study were performed under pseudo-first-order conditions, i.e. [CH_2_OO] ≪ [RCN]. Because of the small absorption cross-section of nitriles at 193 nm (≤1.0 × 10^–22^ cm^2^ molecule^–1^, estimated from available data^37^) and low laser fluence (∼25 mJ cm^–2^) used, the photolysis of reagents were negligible (less than 1 parts-per-million) in the current measurements. Even at the highest [CH_3_CN] used, , the concentration of byproducts of the photolysis at 193 nm was , which could not have any significant effect on the current measurements. In Figure 3, the obtained pseudo-first-order decay rate coefficients (*k*_obs_) of CH_2_OO are shown as a function of [HCN]. The complete results and conditions of the measurements are shown in Table 1. The bimolecular rate coefficient *k*_r_(CH_2_OO + HCN) is obtained from the slope of the equation *k*_obs_ = *k*_loss_ + *k*_r_(CH_2_OO + HCN) × [HCN] fitted to the data, while the intercept reflects the *k*_loss_. The resulting bimolecular rate coefficient for CH_2_OO + HCN reaction is (2.22 ± 0.65) × 10^–14^ cm^3^ molecule^–1^ s^–1^.

**Figure 4:**
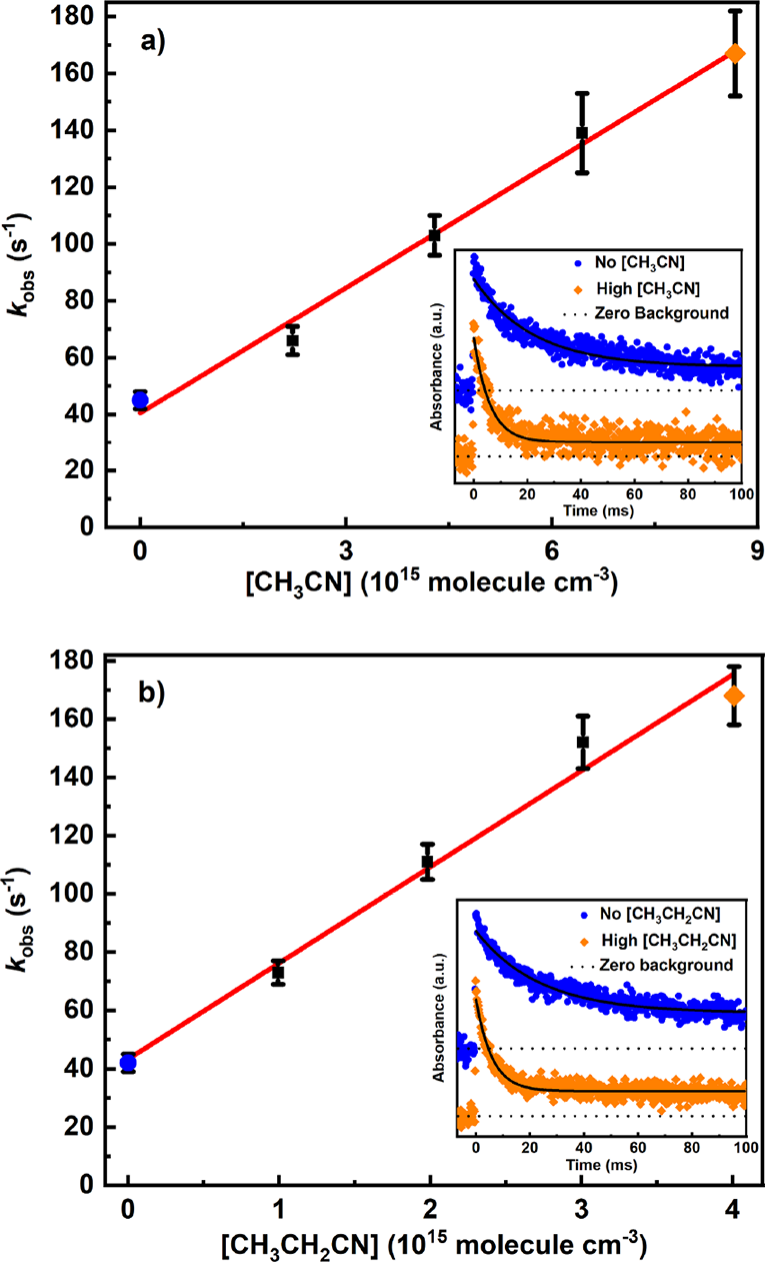
Bimolecular plots of (a) CH_2_OO + CH_3_CN reaction at 320 K and 10.8 Torr. (b) CH_2_OO + CH_3_CH_2_CN reaction at 233 K and 7.9 Torr utilizing CH_2_ICl photolytic precursor. In both panels, the [CH_2_OO] traces in the absence (blue) and presence (orange) of nitrile reagents are shown in the bottom right corners and the colored symbols (blue circles and orange diamonds) depict the measurements that correspond to the shown traces. The traces are shifted vertically for clarity.

Figure 4 shows typical bimolecular plots for CH_2_OO + CH_3_CN and CH_2_OO + CH_3_CH_2_CN reactions. The measured bimolecular rate coefficients for these reactions are shown in Tables 2 and 3 as a function of temperature along with experimental conditions and statistical 2σ experimental uncertainties. Nitrile reactant (CH_3_CN or CH_3_CH_2_CN) dimer concentration in the reactor was investigated using the available monomer–dimer equilibrium data for CH_3_CN.^38^ Extrapolating the data of Renner and Blander^38^ to room temperature, we estimated the maximum gas-phase [(CH_3_CN)_2_] after the bubbler to be less than 1.3% (see the Supporting Information). This has been taken into account in the given nitrile reactant concentrations. Even at the coldest experimental temperature of 233 K (and low pressures), the already low [(CH_3_CN)_2_] dissociated further to monomers after mixing the reactant gas flow with the main gas flow. Due to this high dilution, monomers remained monomers and the final low dimer concentration (‰) in the reactor had negligible effect on the kinetic measurements. Estimated overall uncertainties in the measured rate coefficients are about ±20%. This estimate consists of several sources of uncertainty. The main source of uncertainty is the uncertainty in the employed nitrile concentration due to uncertainties in the saturation vapor pressure of nitrile reagent at the temperature used and flow rates of the mass flow controllers. Uncertainties in the measurement/calibration of reaction zone temperature and pressure also have an effect. The uncertainties associated with the returned parameters from the fittings also cause uncertainty.

**Figure 5:**
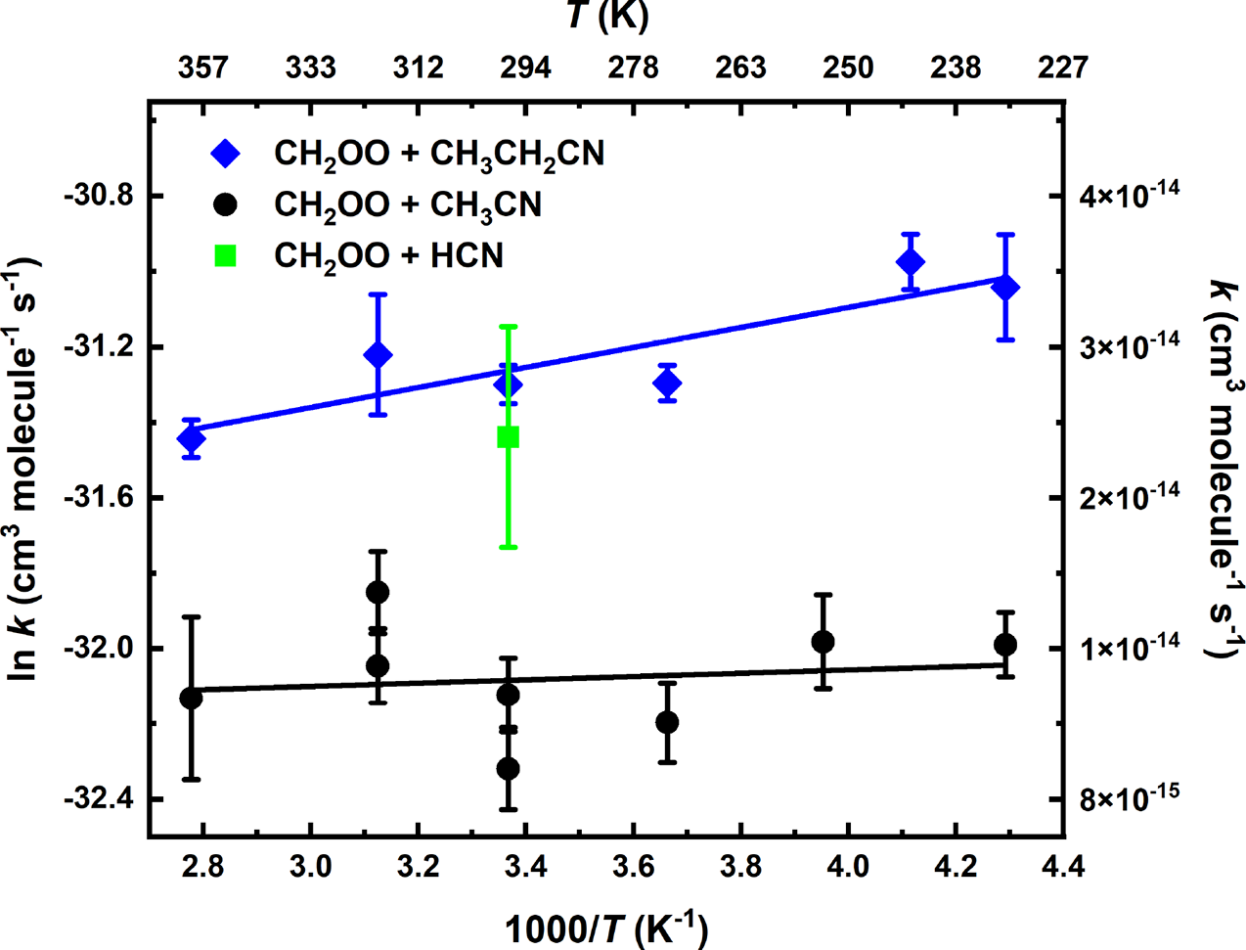
Arrhenius plots of the bimolecular rate coefficients of the CH_2_OO + CH_3_CN (black circles) and CH_2_OO + CH_3_CH_2_CN (blue diamonds) reactions measured in this work at total density of 3.3 × 10^17^ molecules cm^–3^ utilizing CH_2_ICl photolytic precursor. The statistical uncertainties shown are 2σ. Solid lines are the linear least-squares fits to the data. The single bimolecular rate coefficient of CH_2_OO + HCN reaction also measured in this work utilizing CH_2_ICl photolytic precursor is presented with a green square.

The obtained bimolecular rate coefficients for the reactions of CH_2_OO with HCN and CH_3_CH_2_CN are similar at 296 K, with *k*_r_(CH_2_OO + HCN) = (2.22 ± 0.65) × 10^–14^ cm^3^ molecule^–1^ s^–1^, and *k*_r_(CH_2_OO + CH_3_CH_2_CN) = (2.55 ± 0.13) × 10^–14^ cm^3^ molecule^–1^ s^–1^, while the rate coefficient for CH_2_OO + CH_3_CN reaction is around a factor of 2 smaller, *k*_r_(CH_2_OO + CH_3_CN) = (1.02 ± 0.10) × 10^–14^ cm^3^ molecule^–1^ s^–1^. The measured rate coefficient (at 296 K and 10 Torr) for the CH_2_OO + CH_3_CN reaction in this work is in good agreement with the high-pressure-limit value of 1.16 × 10^–14^ cm^3^ molecule^–1^ s^–1^ at 298 K recently calculated by Zhang et al. in their theoretical study.^20^ The CH_2_OO + RCN reactions are faster than the reaction with water monomer, *k*_r_(CH_2_OO + H_2_O) ∼ 10^–16^ cm^3^ molecule^–1^ s^–1^,^39^ but much slower than reactions with carboxylic acids, *k*_r_(CH_2_OO + RCOOH) ∼ 10^–10^ cm^3^ molecule^–1^ s^–1^,^22^ with SO_2_, *k*_r_(CH_2_OO + SO_2_) ∼ 10^–11^ cm^3^ molecule^–1^ s^–1^,^40^ and with water dimer, *k*_r_(CH_2_OO + (H_2_O)_2_) ∼ 10^–12^ cm^3^ molecule^–1^ s^–1^.^41^ Comparing the kinetics of the smallest sCI + nitrile reactions measured in this work with other five-membered-ring forming systems, the bimolecular rate coefficients of CH_2_OO + nitrile reactions are about factor of 10 faster than with alkenes, *k*_r_(CH_2_OO + alkene) ∼ 10^–15^ cm^3^ molecule^–1^ s^–1^ at 298 K,^42,43^ but significantly slower than kinetics with aldehydes and ketones at room temperature, *k*_r_(CH_2_OO + aldehyde/ketone) = (2–10) × 10^–13^ cm^3^ molecule^–1^ s^–1^.^44,45^ The current results show that the CH_2_OO + CH_3_CH_2_CN reaction has a negative temperature dependence, while the CH_2_OO + CH_3_CN reaction is temperature independent within the experimental uncertainty. The theoretical study by Zhang et al.^20^ suggests that the CH_2_OO + CH_3_CN reaction possesses a small negative temperature dependence. The least-squares fits to the linear Arrhenius plots presented in Figure 5 give expressions 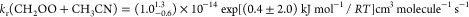
and 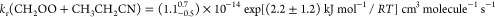
respectively, with 2σ standard fitting uncertainties.

For comparison, the bimolecular rate coefficient of the CH_2_OO + CH_3_CN and CH_2_OO + CH_3_CH_2_CN reactions was also measured at a few temperatures using bromoiodomethane (CH_2_IBr) and diiodomethane (CH_2_I_2_) precursors. The results are presented in Tables 2 and 3, which show that the outcomes of all precursor–photolysis wavelength combinations are in agreement with each other. This, together with the CH_2_OO–precursor dependence measurements shown in Table S1, indicates that the possible products and byproducts of CH_2_ICl precursor photolysis at 193 nm had no effect on the results of the current measurements.

To determine potential pressure dependence of the reactions, we measured the bimolecular rate coefficients of the CH_2_OO + CH_3_CN and CH_2_OO + CH_3_CH_2_CN reactions as a function of nitrogen density at room temperature (296 K). Table 4 shows the obtained results for CH_2_OO + CH_3_CN and CH_2_OO + CH_3_CH_2_CN reactions along with experimental conditions and statistical 2σ experimental uncertainties. The reactions appear to be pressure independent over the range between 10 and 200 Torr, especially once considering the uncertainty of the measurements. The current kinetic measurements of the CH_2_OO + CH_3_CN and CH_2_OO + CH_3_CH_2_CN reactions over wide atmospherically relevant temperature and pressure ranges may suggest (but do not show) that the CH_2_OO + HCN reaction may behave similarly and show only a weak temperature dependence and no dependency on pressure.

**Table 4:** Obtained Bimolecular Rate Coefficients, *k*_r_ (cm^3^ molecule^–1^ s^–1^), and the Conditions Used for the Reaction of CH_2_OO with Nitriles As a Function of Pressure at 296 K. Concentrations Are Presented in molecules cm^–3^^a^

*T*(K)	[N_2_]/10^18^	*p*(Torr)	[CH_3_CN]/10^15^	*k*_loss_(s^–1^)	*k*_r_/10^–14^
CH_2_OO + CH_3_CN Reaction					
296	0.33	10	2.14–8.64	39 ± 2	0.92 ± 0.10
296	0.33	10	2.22–8.91	46 ± 6	1.12 ± 0.11
296	1.6	50	1.87–7.56	28 ± 2	0.92 ± 0.13
296	3.3	100	2.06–8.36	26 ± 3	0.82 ± 0.10
296	6.5	200	2.03–8.58	37 ± 2	0.92 ± 0.10
CH_2_OO + CH_3_CH_2_CN Reaction					
296	0.33	10	1.02–4.41	39 ± 3	2.55 ± 0.13
296	1.6	50	1.03–4.28	14 ± 1	1.83 ± 0.16
296	3.3	100	1.07–4.35	22 ± 1	2.15 ± 0.15
296	6.5	200	1.07–4.36	21 ± 1	2.19 ± 0.10

a CH_2_ICl precursor concentration used: <1.0 × 10^12^ molecule cm^–3^ Estimated initial CH_2_OO concentration <1.0 × 10^11^ molecule cm^–3^. The fixed O_2_ concentration was ∼4.0 × 10^16^ molecule cm^–3^.

### Computational Results

#### Quantum Chemical Results

**Figure 6:**
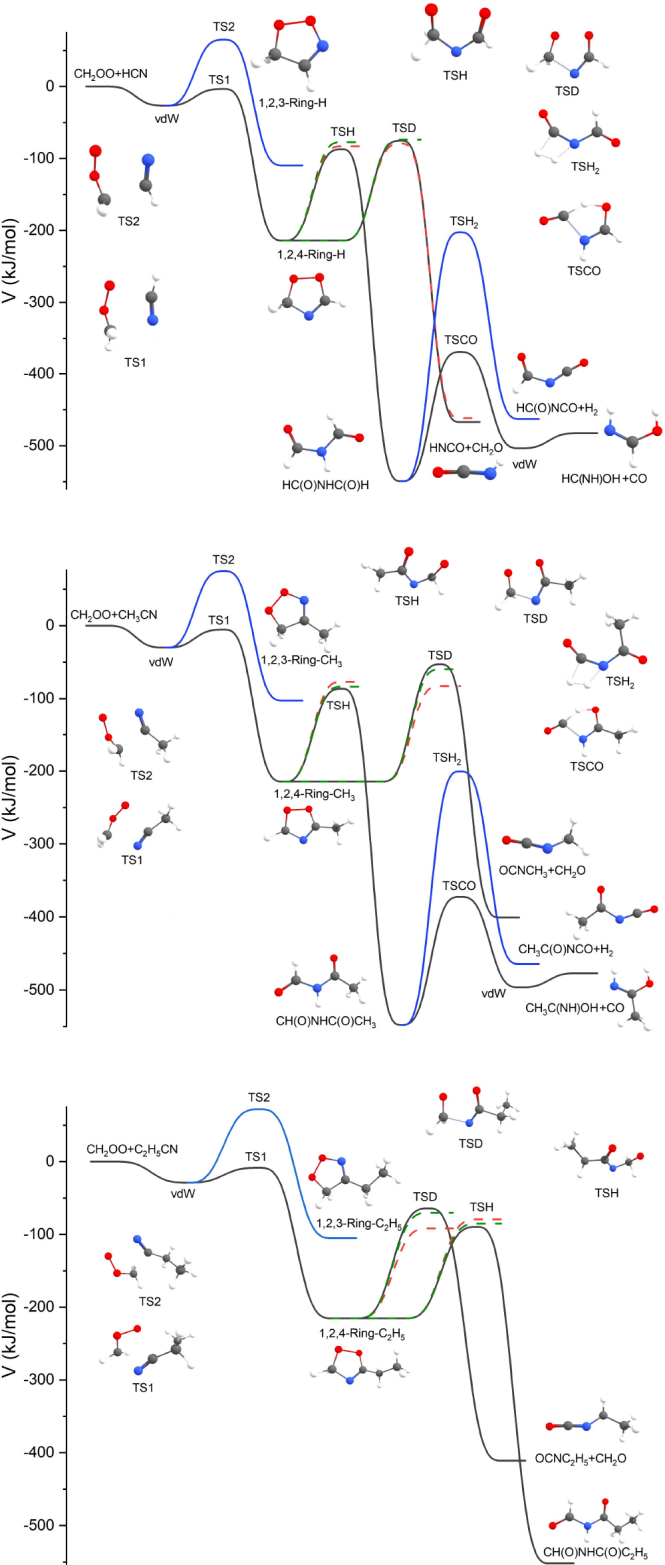
Zero-point-energy corrected potential energy surfaces of the CH_2_OO + RCN reactions. The black line represents the CCSD(T)-F12 energies, the red dotted line the XMC-QPDT2 energies, and the green dotted line the SF-TDDFT energies. For clarity, the latter two are only represented relative to the 3(*R*)-1,2,4-dioxazole. The blue line represents an alternative ring association reaction that does not occur in atmospheric conditions.

As already mentioned, after the barrierless initial association, the lowest energy channel of CH_2_OO + RCN reaction proceeds over the submerged barrier to form a five-membered ring, see Figure 6. Depending on the nitrile reactant, the ring-product is a 1,2,4-dioxazole, 3-methyl-1,2,4-dioxazole, or a 3-ethyl-1,2,4-dioxazole, structurally reminiscent of a secondary ozonide formed in a sCI + carbonyl reaction. We will refer to this intermediary product as ’the dioxazole’ for short. An alternative ring closure reaction, resulting in the formation of a 4(*R*)-1,2,3-dioxazole (pictured in Figure 6 as well as in Figure S2 in the Supporting Information), was also considered. The barrier energy for this pathway was found to be on average 76 kJ mol^–1^ higher than for the main pathway, which is enough to conclude that this reaction does not occur in atmospheric conditions nor did play any role in the current experiments. The dioxazole has two competing unimolecular reactions: a simultaneous ring opening and H-shift resulting in a *N*-formyl(R)amide (R = formyl, acetyl, propionyl) (from now on, ’the rearrangement pathway’) and decomposition into formaldehyde and an isocyanate molecules containing the R functionality (’the decomposition pathway’). Decomposition of *N*-formyl(R)amide was considered for the formyl formamide and formyl acetamide intermediates. Two pathways were found for both. The first is an ejection of a CO group, leaving behind a formimidic acid or acetamidic acid molecule, respectively. The second is an ejection of a H_2_, resulting in formyl isocyanate or acetyl isocyanate. The full reaction pathway, along the reaction potential energy surface presented in Figure 6, is given in Scheme R6.
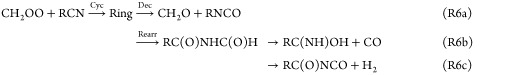
R6a

The chemical similarity of the dioxazole to the secondary ozonide suggests an analysis of the mechanism of ring formation in light of a recent theoretical study on the bimolecular reactions of CH_2_OO with carbonyls.^46^ Wang et al. postulate that the reaction occurs in two steps, the breaking of the C=O π-bond and the ring formation, further noting that the rate-limiting step of the total reaction depends on the carbonyl compound in question. In our studies, we found only one transition state, in which the C≡N bond length is 1.165 Å on average. This is much closer to the nitrile bond length before initial association (1.14 Å) than it is to the C=N bond length in the optimized dioxazole structure (1.27 Å), indicating that breaking of the π-bond is the rate-limiting step in reactions R1–R3).

Out of the two unimolecular reactions found for the dioxazole, the rearrangement pathway corresponds well to the unimolecular pathway found for the five-membered ring formed from the bimolecular reaction between CH_2_OO and C_2_H_2_.^21^ As described by Sun et al., the reaction is well described using one transition state (TSH in Figure 6). The competing decomposition pathway roughly corresponds to one of those found by Jalan et al. for the secondary ozonide;^47^ its transition state corresponds to a simultaneous breaking of the C–N and O–O bonds (TSD in Figure 6), something seen particularly clearly in the motion along the imaginary vibrational mode of the XMC-QDPT2 transition state geometries. The ’immediate’ products of this ring opening reaction are formaldehyde and a highly unstable RN·CO· biradical, which immediately rearranges into the RNCO. The instability of this structure results in imaginary frequencies unusually high for heavy atom motion (−999.8 cm^–1^ for the CH_2_OO + HCN system), meaning that this pathway is slightly favored by tunneling. As for the decomposition reactions of the N-formyl(R)amide, both reactions correspond well to the decomposition pathways found for formamide by Gahlaut et al.^48^ In contrast to those results, for *N*-formyl(R)amide the CO ejection is clearly the dominant decomposition pathway. The barrier energies of the ring opening reactions are found in Table 5, and visualizations of all transition states are found in Figure S2 in the Supporting Information.

As seen from Figure 6, the dioxazole may have a significant chemical activation, about 200 kJ mol^–1^, immediately after its formation. This additional energy may result in further isomerization and/or the decomposition of the dioxazole. Product yields from the ME simulation were used to assess the stabilization and further reactions of the dioxazole intermediate. The barrier energies for further reactions of the dioxazole are presented in Table 5. As seen in these results, the single-reference and multireference calculations agree reasonably well on the rearrangement barrier, but not so much on the decomposition barrier, especially for the two larger systems. Notably, the XMC-QDPT2 energies are considerably lower than either the CCSD(T) or SF-TDDFT results. A detailed comparison of the optimized geometries is found in the Supporting Information; in summary, there is a reason to assume that the XMC-QDPT2 barrier energies are the most accurate for the decomposition reaction. However, we caution that the fairly large differences between the coupled-cluster and XMC-QDPT2 energies indicate a quite high overall uncertainty for the energetics of this pathway, which, consequently, results in a higher than anticipated uncertainty in the ME product yields.

Note also the decreasing Δ*E*_XMC_ trend in regards to the molecule size, presumably due to the longer alkyl substituent stabilizing the intermediary ·OCN·R biradical. This trend can be assumed to continue for larger RCN reactants. For the rearrangement transition state, no separate XMC-QDPT2-level geometry was found. The presented energies for TSH are thus single-point energies calculated on the ωB97X-D geometries. Nevertheless, all three methods used to calculate the energy are in good agreement for this reaction, so presumably the multiconfigurational character of the O–O scission does not interfere with the accuracy of the CCSD(T) results. This is consistent with the findings of Sun et al. that the H-shift requires slightly more energy than the O–O scission.^21^

#### Master Equation Simulation Results

ME simulations were performed utilizing all three potential energy surfaces: CCSD(T), XMC-DPT2, and SF-TDDFT. Computational rate coefficients were determined using Bartis-Widom vector analysis.^49^ The rate coefficients were obtained with the pseudo-first-order expression *k*_r_[RCN] = λ, λ being the Bartis-Widom eigenvalue. The results are shown in Table 6. As seen from the results in the Table 6, the computed rate coefficients agree with the experimental rate coefficients relatively well, within about factor of 3, without any tuning of energies or other parameters. This is taken as a clear indication that the modeled reaction mechanism indeed corresponds to the measured reaction.

**Table 5:** Barrier Energies of the Competing Dioxazole Reactions, Presented in kJ mol^–1^^a^

	Δ*E*_CC_	Δ*E*_XMC_	Δ*E*_SF_
Decomposition			
HCN	138.7	134.8	140.2
CH_3_CN	161.7	131.8	154.8
C_2_H_5_CN	151.2	123.7	145.2
H-shift			
HCN	126.9	131.0	136.8
CH_3_CN	128.2	137.7	131.0
C_2_H_5_CN	125.8	136.0	130.1

a The subscripts CC, XMC, and SF refer to the CCSD(T), XMC-DPT2, and SF-TDDFT levels of theory, respectively.

**Table 6:** Comparison of Computationally and Experimentally Determined Bimolecular Rate Coefficients^a^

	HCN		CH_3_CN		C_2_H_5_CN	
	*k*_r_ (Comp.)	*k*_r_ (Exp.)	*k*_r_ (Comp.)	*k*_r_ (Exp.)	*k*_r_ (Comp.)	*k*_r_ (Exp.)
*T* (K)						
233			4.13	1.28	8.0	3.30
253			3.46	1.29	6.2	3.53
273			2.98	1.04	5.0	2.56
296	4.65	2.22	2.55	1.02	4.0	2.55
320			2.30	1.34	3.4	2.76
360			1.99	1.11	2.9	2.21
*p* (torr)						
40			2.66	0.92	4.04	1.83
100			2.67	0.82	4.06	2.15
200			2.68	0.92	4.06	2.19
250	4.65	2.22				

a All *k*_r_ are presented in values of (10^–14^ cm^3^ molecule^–1^ s^–1^). These ME calculations are performed using the XMC-QDPT2 energies.

**Figure 7:**
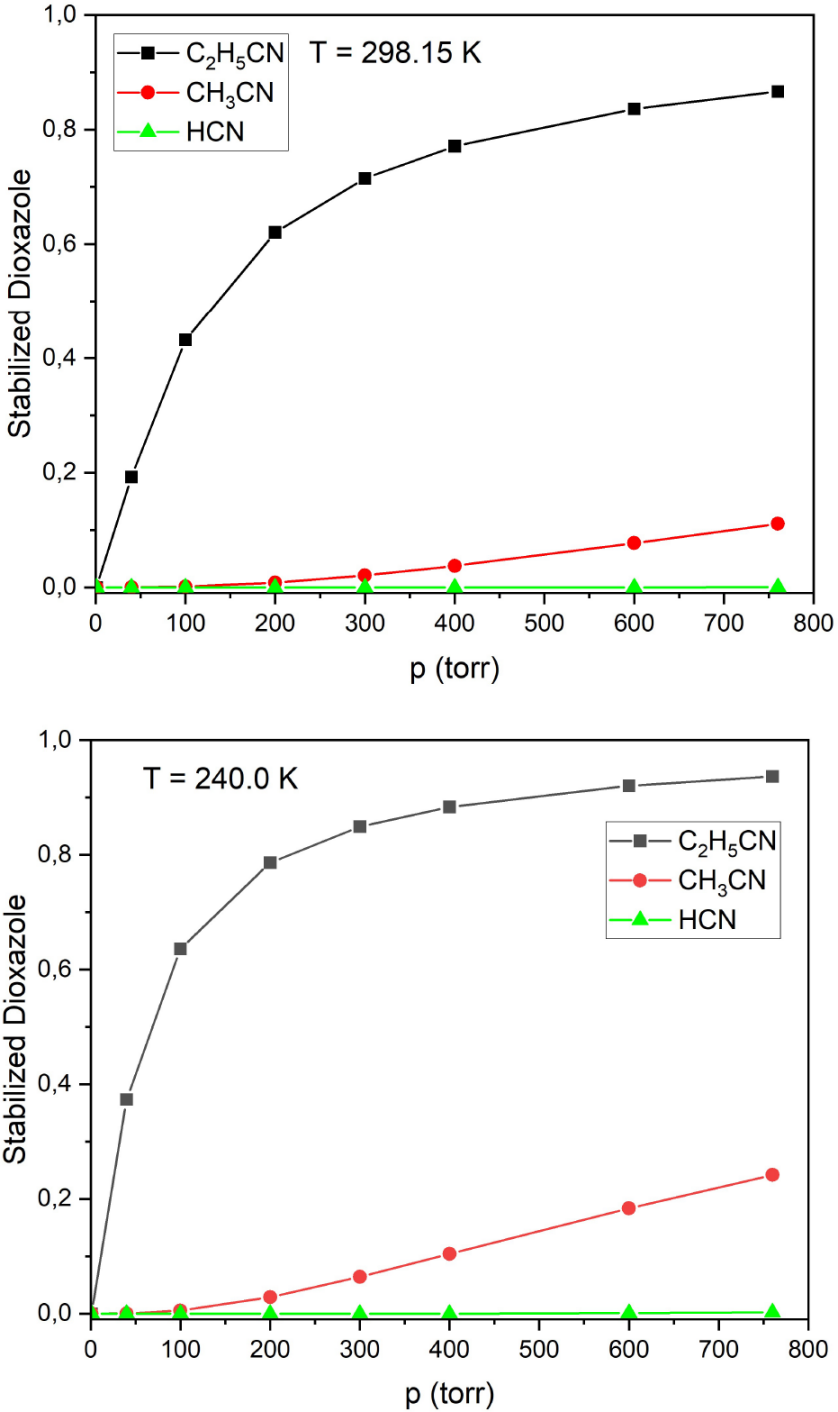
Dependence of the fraction of stabilized dioxazole on pressure at *T* = 298.15 K (above) and *T* = 240.00 K (below), calculated using the XMC-QDPT2 energies.

**Figure 8:**
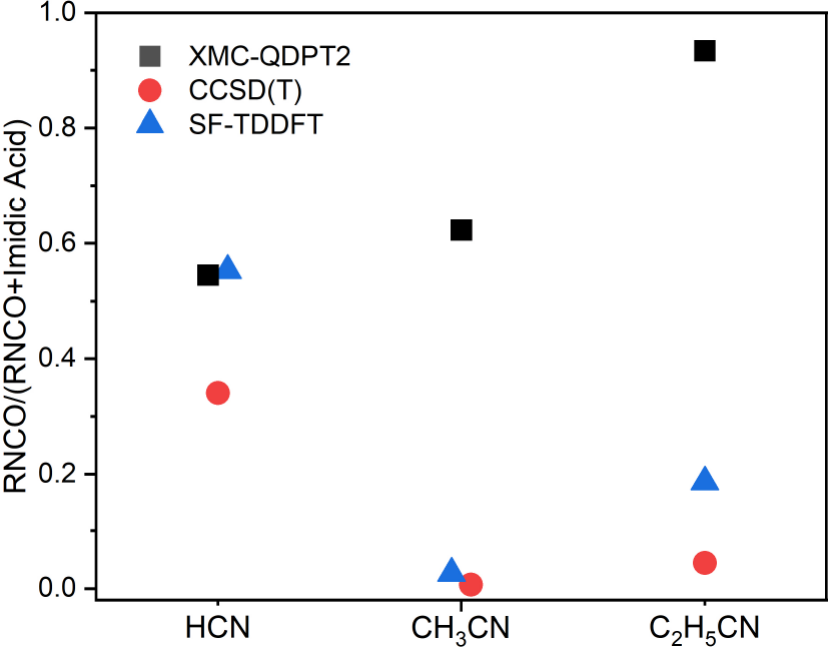
A comparison of isocyanate yield from the chemically activated decomposition of dioxazole simulated using different potential energy surfaces for reactions R1–R3. The data for HCN and CH_3_CN are shifted along the horizontal axis for clarity.

A comparison of the product yields from the ME simulations of the reactions R1–R3 using the all three potential energy surfaces are presented in full in the Supporting Information. The most important findings are summarized in Figure 7, which presents the ME simulated yield of the thermalized dioxazole as a function of pressure at two temperatures, and in Figure 8, which presents the branching ratio of the two important chemically activated decomposition channels in relation to each other. From Figure 7 we observe that the two chemically activated reaction channels dominate overwhelmingly over dioxazole stabilization in the CH_2_OO + HCN reaction and to a lesser extent in the CH_2_OO + CH_3_CN system, whereas for the larger CH_2_OO + C_2_H_5_CN system the stabilized dioxazole is overwhelmingly the main product at atmospheric pressure. At lower pressures the stabilization is less efficient, meaning that the chemically activated reactions also dominate for the CH_2_OO + C_2_H_5_CN system. The simulated yields of the decomposition (resulting in CH_2_O + RNCO) and rearrangement (resulting in the RC(O)NHC(O)H intermediate) channels vary between the three potential surfaces, see Tables S7–S9 in the Supporting Information. The decomposition reaction is obviously favored by entropy, and it is the major pathway on the XMC-DPT2 surface, where the two reactions have similar barrier energies. The SF-TDDFT and CCSD(T) potential surfaces, where the decomposition barrier is noticeably higher for the two larger systems (R2) and (R3) (see Figure 6), tip the balance in favor of the rearrangement pathway. The RC(O)NHC(O)H does not show any stabilization even at atmospheric pressure, but further decomposes into RC(NH)OH and CO as shown in Scheme R6b.

### Atmospheric Relevance

As already discussed in the introduction section, the most important gas-phase sink reaction for nitriles in the atmosphere is reaction with OH. With an assumed average OH radical concentration of 10^6^ molecules cm^–3^ in the atmosphere,^51^ the lifetime of HCN in relation to reaction with OH is  3.5 years. An estimated total [sCI] in boreal forest environment is 5 × 10^4^ molecules cm^–3^,^50^ of which a large fraction presumably originates from CH_2_OO. Using these values, we may compare the atmospheric lifetime of nitriles with regard to these two reactions, see Table 7. The results imply that for nitriles the reaction with sCI is, on average, a minor sink at most. However, reaction with sCI might be a significant local sink for nitriles well above the sea level, particularly in rising fire plumes, as these are a net source of not only nitriles, but also ozone and alkenes, the reactants required to produce sCIs.^52^

**Table 7:** Comparison of the RCN Lifetime with Regard to Reaction with OH and CH_2_OO^a^

	τ_OH_	τ_CH_2_OO_	τ_CH_2_OO_/τ_OH_	τ_tot_
HCN	3.5 years	29 years	8.1	4.4 months^16^
CH_3_CN	0.8 years	62 years	78.4	Unknown^17^
C_2_H_5_CN	3.0 months	25 years	99.6	Unknown

a OH reaction rates are from refs (14 and 15). CH_2_OO + RCN rates are from this work. Note that τ_CH_2_OO_ is calculated using the total rate of CIs rather than [CH_2_OO] specifically,^50^ and that the presented value for τ_tot_ is largely determined by ocean uptake rather than chemical reactions.

What are the products of reactions R1–R3 in the atmospheric conditions? From the full ME results presented in Tables S7–S9 in the Supporting Information, one finds that the simulations using different quantum chemical methods agree fairly well on the stability of the dioxazole. We may thus claim with a reasonable confidence that the dioxazole ring is the main product of the CH_2_OO + C_2_H_5_CN reaction, and most likely also for reactions with larger nitriles, though these reactions may play a limited role in their atmospheric degradation. This is because larger RCN compounds have shorter atmospheric lifetimes^14^ due to their fast reactions with OH radicals. For the CH_2_OO + HCN and CH_2_OO + CH_3_CN reactions, the main products are either CH_2_O and RNCO or RC(NH)OH and CO. As discussed in Quantum Chemical Results section, the branching ratio between these product channels has a large uncertainty. The imidic acid (RC(NH)OH) is likely to rearrange into its more stable amide tautomer (RC(O)NH_2_) with any remaining chemical activation.

Further degradation kinetics and mechanisms of the stabilized dioxazole formed in reactions R2 and R3 as well as in reactions of larger nitriles are of interest. The stabilized dioxazole should be stable with respect to unimolecular decomposition, since even the lowest barrier calculated in this work for decomposition, see Table 5, is about 124 kJ/mol (about 30 kcal/mol). An in-depth analysis of potential bimolecular reactions and kinetics of the stabilized dioxazole with atmospheric constituents is outside the scope of this work. However, we can make some (very) rough estimates by assuming that the stabilized dioxazole has a similar bimolecular reactivity to secondary ozonides. For secondary ozonides, the barrier energies of the bimolecular reactions with NH_3_, H_2_O, and (H_2_O)_2_ are high enough to effectively rule these reactions out.^53^ It may appear that finally OH radical reaction with the stabilized dioxazole is the main degradation mechanism. Peeters et al.’s SAR suggests the rate coefficient *k*_OH_ = 8.5 × 10^–11^ cm^3^ molecule^–1^ s^–1^ for an alkyl-substituted cyclopentene,^54^ which is a sum of rate coefficients for both carbons partaking in the C=C bond. For OH + stabilized dioxazole reaction, the OH addition rate is likely to be faster for addition to the imine carbon due to its electropositivity, but lower for addition to the nitrogen due to the instability of the carbon centered radical.^55^ Hydrogen-abstraction from the CH_2_ group(s) is a viable bimolecular OH radical reaction channel, since it leads to the formation of resonance-stabilized radical(s). We estimate a total bimolecular rate coefficient about 10^–11^ cm^3^ molecule^–1^ s^–1^ for the OH + stabilized dioxazole reaction, leading to dioxazole lifetime of a few days in the atmosphere.

## Conclusions

In this work, we have measured kinetics of the smallest stabilized Criegee intermediate (CH_2_OO) with the three smallest nitriles (HCN, CH_3_CN, and CH_3_CH_2_CN) at temperatures between 233 and 360 K using the transient UV-absorption spectroscopy method. In the experiments, we utilized, for the first time, a new photolytic precursor for production of formaldehyde oxide, chloroiodomethane (CH_2_ICl), the photolysis of which at 193 nm in the presence of O_2_ produces CH_2_OO. This new method enables kinetic measurements of CH_2_OO at much lower atmospherically relevant temperatures than has been possible before. The kinetic results show that CH_2_OO reacts with nitriles with rate coefficients of (0.8–3.5) × 10^–14^ cm^3^ molecule^–1^ s^–1^. Kinetics of CH_2_OO reactions with nitriles are thus about ten times faster than reactions with alkenes, but reactions of CH_2_OO with aldehydes and ketones are about ten times faster than reactions with nitriles. The measured bimolecular rate coefficient of the CH_2_OO + HCN reaction is (2.22 ± 0.65) × 10^–14^ cm^3^ molecule^–1^ s^–1^ at 296 K and 250 Torr. The CH_2_OO + CH_3_CH_2_CN reaction was found to have a weakly negative temperature dependency with an Arrhenius activation energy −2.2 ± 1.2 kJ mol^–1^, while the CH_2_OO + CH_3_CN reaction was observed to be temperature independent within the experimental uncertainty. The measurements show that the kinetics of CH_2_OO + CH_3_CN and CH_2_OO + CH_3_CH_2_CN reactions are independent of pressure over the range between 10 and 200 Torr of nitrogen at 296 K. This suggests that kinetics of CH_2_OO + HCN reaction is also likely independent of pressure under atmospherically relevant conditions. The experimental kinetic results imply that the CH_2_OO + RCN reactions are not a major atmospheric sink for nitriles.

Our computational studies successfully explain the results of the current kinetic measurements. The CH_2_OO + RCN is a barrierless reaction (with respect to the free reactants) with a submerged energy barrier leading to a five-membered-ring formation, resulting in a 3(*R*)-1,2,4-dioxazole. Our master equation model shows that the five-membered-ring formation is followed (at least for HCN and CH_3_CN nitriles) by its chemically activated decomposition into formaldehyde and an isocyanate, or, by a rearrangement, into a *N*-formyl(R)formamide, which is then decomposed into carbon monoxide and an imidic acid. The relative importance of these two reactions is difficult to conclusively judge based on our simulations. Either way, both of these product channels dominate over the dioxazole formation at lower pressures, which suggests these product channels may play an important role at higher tropospheric altitudes and in the stratosphere.

## Supporting Information Available

Additional experimental and computational details: precursor spectrum and precursor effect on measurements; dimerization of nitrile reagents; description of quantum chemical methodology; reliability of single-reference calculations; comparison of single-reference and multireference geometries; product distibution data (from ME calculations); Criegee + nitrile association rate; calculation of Lennard-Jones parameters; sensitivity analysis of energy transfer model (of ME simulations); comparison of RRKM and ILT results (PDF)
Output files of the quantum chemical ωB97X-D/aug-cc-pVTZ optimization and frequency calculations and CCSD(T)-F12a/VDZ-F12 single point energy calculations, as well as the MESMER 6.0 input and output files for the ME simulations (ZIP)
