# Supplementary material for: An Experimental and Master Equation Investigation of Kinetics of the CH2OO + RCN Reactions (R = H, CH3, C2H5) and Their Atmospheric Relevance
Source: J Phys Chem A. 2023 Jan 5;127(2):477–88. doi: 10.1021/acs.jpca.2c07073 (PMC9869398; doi:10.1021/acs.jpca.2c07073)
Supplement: Supplementary file 1 — jp2c07073_si_001.pdf [file jp2c07073_si_001.pdf]

Supporting Information: An Experimental and Master-Equation Investigation of Kinetics of the CH_2OO + RCN Reactions (R = H, CH_3, C_2H_5) and their Atmospheric Relevance

Lauri Franzon[a], Jari Peltola[a], Rashid Valiev, Niko Vuorio, Theo Kurtén, and Arkke Eskola*

Department of Chemistry, University of Helsinki,

P.O. Box 55 (A.I. Virtasen aukio 1), 00014 Helsinki, Finland

E-mail: arkke.eskola@helsinki.fi

[0a] *These authors contributed equally to this work*

Experimental Supplementary Information

Precursor spectrum and precursor effect on measurements

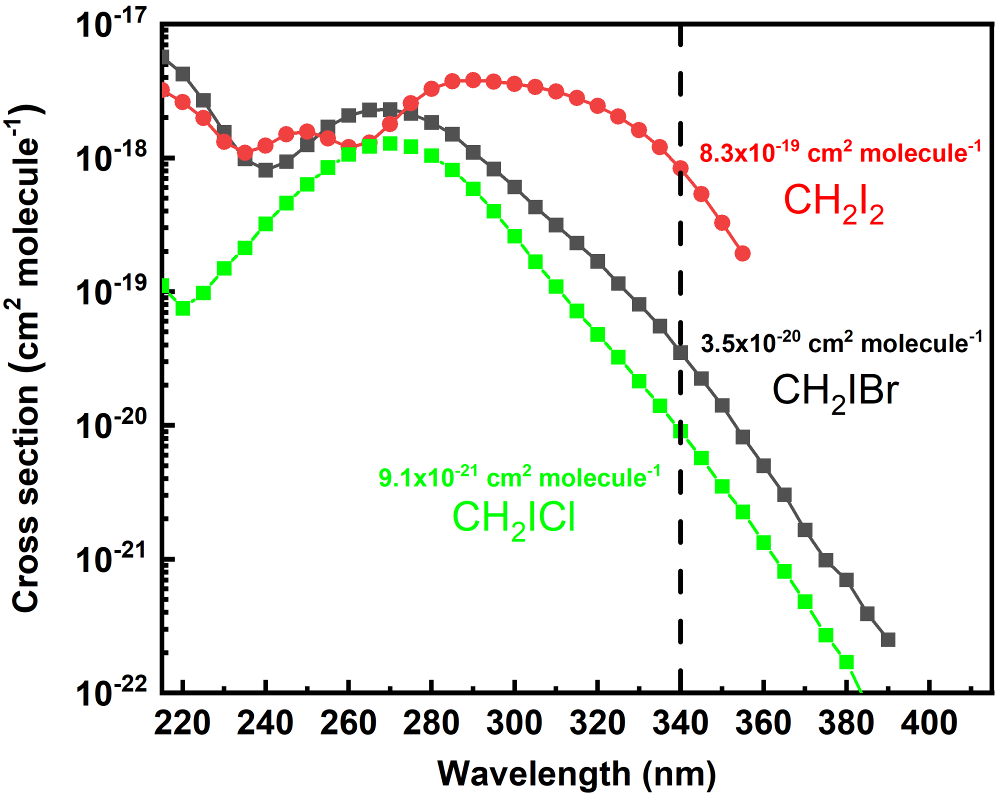

Figure S1: The absorption cross-sections of CH_2I_2, CH_2IBr, and CH_2ICl precursors as function of wavelength.

The UV absorption cross-section of gaseous CH_2ICl as a function of wavelength is presented in Fig. S1.[S1,S2] In addition, Fig. S1 shows the cross-sections of CH_2I_2,[S3] CH_2IBr,[S3] and CH_2ICl[S1,S2] photolytic precursors as function of wavelength. CH_2I_2 has about 90 times stronger absorption cross-section than CH_2ICl at 340 nm region, where the absorption of CH_2OO has its maximum.

Results of the experiments with higher precursor concentration, but with lower laser fluence, to test the importance of Criegee-precursor, $CH_2OO + CH_2ICl$ reaction are shown

in Table S1. In the Table S1, the measured decay rate coefficients of CH_2OO (fixed initial $[CH_2OO]$) in the absence of nitrile reagent are shown as function of $[CH_2ICl]$ at 296 K and 10 Torr. The measured decay rate coefficients do not depend on the $[CH_2ICl]$ to any significant extent.

Table S1: Measured decay rate coefficients of CH_2OO as a function of CH_2ICl concentration without nitrile reagent at 296 K and 10 Torr. Concentrations are presented in molecule cm^{-3}. The statistical uncertainties shown are 2σ.

T(K)	$[N_2]/10^{18}$	p(Torr)	$[CH_2ICl]/10^{12}$	laser flux (mJ cm^{-2})	k_{loss}(s^{-1})
296	0.33	10	0.41	20	41 ± 3
296	0.33	10	0.82	10	44 ± 2
296	0.33	10	1.63	5	36 ± 2

The fixed CH_2OO concentration was $< 1.0 \times 10^{11}$ molecule cm^{-3}. The fixed O_2 concentration was $\sim 4.0 \times 10^{16}$ molecule cm^{-3}.

Dimerization of nitrile reagents

The possible dimerization of nitrile reagents in the reactor was investigated using the available dimer equilibrium data for CH_3CN.[S4] Extrapolating the data of Renner and Blander[S4] to 298 K and 233 K (see Table S2), we can estimate the gas-phase $[(CH_3CN)_2]$ in the reactor. The CH_3CN or CH_3CH_2CN nitrile reagent was supplied to the reactor by bubbling nitrogen gas at known pressure and flow rate through a temperature-stabilized liquid nitrile reagent with known vapour pressure at used temperature (typically at 298 K). Assuming that the total CH_3CN pressure (p_T) after the bubbler is the same as its vapor pressure at the temperature in question (88 Torr at 298 K). The total CH_3CN pressure (p_T) is the sum of the partial pressures of monomer (p_M) and dimer (p_D), $p_T = p_M + p_D$. The equilibrium constant K for the reaction $2\,CH_3CN \rightleftharpoons (CH_3CN)_2$ can be defined as

$$K = \frac{p_D}{p_M^2}$$

From the equilibrium constant of the dimerization, the partial pressure of the monomer can be calculated as

$$p_{\mathrm{M}} = \frac{\sqrt{1+4Kp_{\mathrm{T}}}-1}{2K}$$

Assuming that all dimers fully dissociate to monomers in the reactor due to the high dilution, we may express the total partial pressure of monomers in the reactor P_{M} by substituting $p_{\mathrm{D}} = p_{\mathrm{T}} - p_{\mathrm{M}}$:

$$P_{\mathrm{M}} = p_{\mathrm{M}} + 2p_{\mathrm{D}} = 2p_{\mathrm{T}} - p_{\mathrm{M}} = 2p_{\mathrm{T}} - \frac{\sqrt{1+4Kp_{\mathrm{T}}}-1}{2K}$$

Using the extrapolated equilibrium value $K = 0.1153$ at 298 K ($p_{\mathrm{T}} = 88$ Torr), we estimated the maximum gas-phase $[(CH_3CN)_2]$ after the bubbling to be 1.3% ($\frac{p_{\mathrm{D}}}{p_{\mathrm{T}}} = \frac{p_{\mathrm{T}}-p_{\mathrm{M}}}{p_{\mathrm{T}}} = 0.013$). This has been taken into account in the given concentrations. Even at the coldest temperature of 233 K ($K = 1.3067$, $p_{\mathrm{T}} < 0.2$ Torr), all dissociated dimers (high dilution) remain virtually monomers in the reactor and the low dimer concentration ($< 0.5‰$) is negligible in the measurements. Equilibrium data for CH_3CH_2CN dimerization could not be found in the literature, but we assume it to be equivalent or close to CH_3CN data. The partial pressure of HCN sample gas was small (< 0.3 Torr at 760 Torr), thus the dimer concentration of HCN sample was also negligible.[S5]

Table S2: Equilibrium constants for dimer formation of CH_3CN.

T(K)	Measured $K(\mathrm{atm}^{-1})$[S4]	Extrapolated $K(\mathrm{atm}^{-1})$
387.3	0.0158	
372.2	0.0206	
361.2	0.0253	
338.4	0.0405	
298.0		0.1153
233.0		1.3067

The equilibrium constants at temperatures 298 K and 233 K are extrapolated from the measured data of Renner and Blander[S4] using the linearized equilibrium constant equation $\ln K = A/T + B$, where the constants A and B are related to the enthalpy and entropy of the reaction, respectively.

Quantum Chemical Supplementary Information

Description of Quantum Chemical Methodology

The following programs were used for quantum chemical calculations: Gaussian 16[S6] for molecular structure optimization and single-reference energy calculations, Firefly[S7] for XMC-QDPT2 calculations and GAMESS-US[S8] for the TDDFT Spin-Flip calculations.

In the single-reference calculations, molecular geometries were optimized using Density Functionals on the ωB97X-D/aug-cc-pVTZ level of theory, with analytical frequency analysis implemented on the resulting structure. The electronic energies of the DFT-optimized structures were calculated using CCSD(T)-F12/VDZ-F12, whereas for vibrational zero-point energies the ωB97X-D/aug-cc-pVTZ level results were used. For transition state structures specifically, the ωB97X-D/aug-cc-pVTZ optimizations were performed with the spin unrestricted option on in case of spin contamination. Additionally, due to the relative abundance of low-frequency vibrational modes, internal rotor searches were performed on the same level of theory, using the Hindered rotor solver in Gaussian,[S9] which fits the modes to equation 1. As as result, the vibrational modes corresponding to methyl and ethyl rotations in the intermediates of the $CH_2OO + CH_3CN$ and $CH_2OO + C_2H_5CN$ systems most crucial for the final product yield were replaced with Hindered Rotor models. The modes in question are tabulated in Table (S3).

$$V(\theta) = \frac{V}{2}\left(1 - \cos(n\theta)\right) \tag{1}$$

Multi-reference calculations were performed exclusively on the dioxazole and the decomposition and rearrangement transition states. Refer to the section on reliability of single-reference results for the motivation behind this prioritization. The transition state and dioxazole geometries were obtained using the extended quasi-degenerate 2nd-order multireference perturbation theory (XMC-QDPT2),[S10] with the def2-TZVP basis set. We chose

an active space consisting of 10 electrons in 10 molecular orbitals (MOs) for searching for the singlet ground states of both transition state and minima, using the same orbitals for the decomposition pathway. For the rearrangement pathway, no XMC-QDPT2-level transition state was found using this method, and as such single-point calculations based on the ωB97X-D/aug-cc-pVTZ structures was carried out for both transition state (TS) and dioxazole. Note that the active spaces were built from MOs corresponding to the bonding σ-MOs of the O-O and C-N bonds and MO corresponding to the antibonding σ^*-MOs of the same bonds, the several π-bonding MOs of the O-O bonds.

In addition, the Spin-Flip Time Dependent Density Functional Theory (SF-TDDFT) method[S11] was applied to searching of TS and minima for both channels. This was performed on the B3LYP/aug-cc-pVTZ level of theory. Optimized transition states were found for both channels, and the agreement between the methods was good.

Table S3: ωB97X-D/aug-cc-pVTZ level of theory frequencies of the replaced vibrational modes and potential energies of the respective hindered rotors. The barrier energies are expressed in units of kJ mol^{-1} and the vibrational frequencies in units of cm^{-1}. ImH refers to the Imidic acid.

Rotor		TS1	Ring	TSD	TSH	TSCO	ImH
Met (CH_3CN)	V	5.300	3.330	7.040	1.618	2.508	4.558
Met (CH_3CN)	Freq	158.6381	114.9990	177.5463	242.1945	92.5021	128.4110
Met (C_2H_5CN)	V	4.184	8.080	21.008	12.682		
Met (C_2H_5CN)	Freq	226.7572	210.9791	220.3772	213.2741		
Et (C_2H_5CN)	V	12.432	3.642	15.730	4.874		
Et (C_2H_5CN)	Freq	582.2942	47.7546	165.7717	37.0839		

Reliability of Single Reference Calculations

Criegee Intermediates have a notoriously multi-configurational character, and as such the need for multi-reference calculations was evaluated using the CCSD(T)-F12/VDZ-F12 T1 & D1 diagnostics form the Molpro output files. This data is found in Table S4. As you see, quite a few of the values are above the reference values 0.02 for T1 and 0.05 for D1, suggesting

multi-configurational character. However, the diagnostics for the Reactant Complex and the ring-closure Transition State were largely on the same order of magnitude as those of the free CH_2OO. It thus concluded that multi-reference determined energies of these systems would probably not improve the accuracy of the ME model. On the other hand, the difference in diagnostic values between the dioxazole and the two transition states of its reactions is quite considerable. The activation energies of these two reactions are crucial for determining the final atmospheric products, thus these structures were selected for multi-reference calculations.

Table S4: T1 and D1 diagnostics of the CCSD(T)-F12/VDZ-F12 calculations. Each pair of columns is categorized by nitrile substituent R.

System	T1 (H)	D1 (H)	T1 (CH_3)	D1 (CH_3)	T1 (C_2H_5)	D1 (C_2H_5)
CH_2OO	0.041	0.164	0.041	0.164	0.041	0.164
RCN	0.015	0.031	0.013	0.031	0.012	0.032
CH_2OO+RCN	0.032	0.154	0.030	0.155	0.028	0.156
TS1	0.030	0.141	0.028	0.144	0.026	0.144
Ring	0.016	0.051	0.014	0.051	0.014	0.051
TSD	0.044	0.210	0.042	0.223	0.036	0.204
CH_2O+IsoC	0.017	0.049	0.016	0.050	0.015	0.048
Isocyanate	0.018	0.050	0.016	0.048	0.015	0.048
Formaldehyde	0.015	0.042	0.015	0.042	0.015	0.042
TSH	0.024	0.094	0.023	0.100	0.021	0.091
Formylamide	0.016	0.054	0.015	0.054	0.015	0.054
TSCO	0.018	0.059	0.016	0.058		
ImH+CO	0.015	0.043	0.015	0.044		
ImH	0.014	0.042	0.013	0.043		
TSH_2	0.019	0.055	0.018	0.055		
AceIsoC	0.018	0.052	0.017	0.053		

Comparison of DFT and XMC-QDPT2 geometries

The most accurate geometries for each compound are presented in Fig. S2. As seen in the main article, the three different quantum chemical methods agree well with each other on the activation energies of the rearrangement pathways for all three CH_2OO+NCR systems and on the energy of the CH_2OO + HCN decomposition pathway, but not so much for the two larger

systems. Specifically, the XMC-QDPT2 energies were almost 30 kJ mol^{-1} lower than the DFT energies. A comparison of the ωB97X-D/aug-cc-pVTZ and XMC-QDPT2-optimized internal coordinates of the dioxazole ring are presented in Table S5 separately for the stable dioxazole and for the decomposition transition state. The SF-TDDFT geometries are not presented here because they are surprisingly close to the single-reference ωB97X-D/aug-cc-pVTZ geometries. The XMC-QDPT2 geometries however diverge significantly from both DFT geometries, particularly when it comes to the O-O distance. The difference in bond length between the XMC-QDPT2 and ωB97X-D geometries us on average +0.044 Å for the dioxazole and -0.210 Å for the decomposition transition state. One might as such make the conclusion that the excited state contributing to the multi-configurational character of the system involves molecular orbitals centered on the O-O bond. This was taken as indication that the XMC-QDPT2 finds a slightly different reactive path with an earlier saddle point, and thus captures the multi-configurational character of the compounds better than density functional methods.

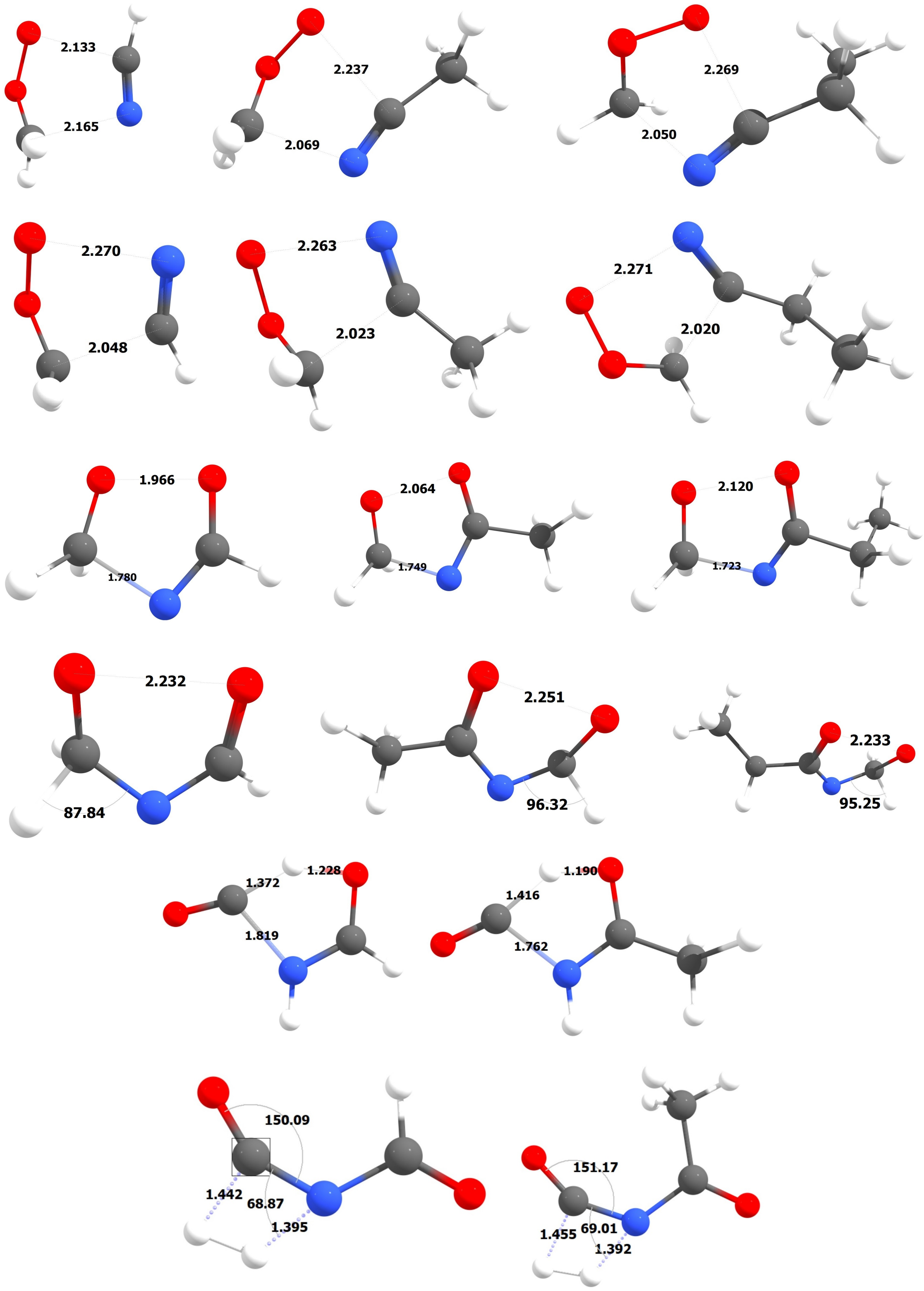

Figure S2: Structures of the transitions states, optimized at the XMC-QPT2 level of theory for the decomposition reaction and at the ωB97X-D/aug-cc-pVTZ level of theory for the other structures. From above: TS1, TS2, TSD, TSH, TSCO and TSH_2.

Table S5: Differences (all in Å) between ωB97X-D/aug-cc-pVTZ- and XMC-QDPT2-optimized internal coordinates in the dioxazole ring structure. SR and MR refer to Single-Reference and Multi-Reference, respectively.

r (Ring)	HCN_{SR}	HCN_{MR}	CH_3CN_{SR}	CH_3CN_{MR}	$C_2H_5CN_{SR}$	$C_2H_5CN_{MR}$	$\langle\Delta r\rangle$
O-O	1.438	1.481	1.434	1.477	1.433	1.477	0.044
O-C(=N)	1.341	1.349	1.350	1.360	1.353	1.360	0.008
O-C(-N)	1.447	1.440	1.448	1.439	1.443	1.438	-0.007
C=N	1.256	1.268	1.261	1.272	1.262	1.273	0.012
C-N	1.441	1.450	1.436	1.446	1.438	1.447	0.009
r (TS)	HCN_{SR}	HCN_{MR}	CH_3CN_{SR}	CH_3CN_{MR}	$C_2H_5CN_{SR}$	$C_2H_5CN_{MR}$	$\langle\Delta r\rangle$
O-O	2.244	1.966	2.300	2.064	2.237	2.120	-0.210
O-C(=N)	1.211	1.249	1.210	1.248	1.237	1.240	0.026
O-C(-N)	1.253	1.278	1.253	1.278	1.253	1.278	0.024
C=N	1.281	1.282	1.281	1.282	1.281	1.282	-0.009
C-N	1.735	1.780	1.735	1.780	1.735	1.780	-0.024

Master Equation Supplementary Information

The exact energies of all the compounds used in the ME model of the single-reference potential surface are presented in Table S6. TS2 is included in the former for completion despite not being included in the ME due to not being relevant. Multi-reference results were only calculated for the unimolecular reactions of the 3(R)-1,2,4-dioxazole, and those energies are already presented in the main article in Table 6.

Product Distibution Data

The product yields of all the ME simulations varying pressure and temperature are presented in tables S7, S8 and S9 separately for all three values of the dioxazole decomposition barrier energies. The results are discussed in the main article.

Table S6: Energies (CCSD(T) unless stated otherwise) for all compounds included in the ME, presented relative to the energy of CH_2OO+RCN in kJ mol^{-1}. vdW again refers to a van der Waals complex and ImH to the Imidic acid.

	HCN	CH_3CN	C_2H_5CN
$CH_2OO + RCN$	0	0	0
vdW	-26.4356	-30.1145	-28.7732
TS1	-3.3297	-5.4589	-8.4837
TS2	+65.1605	+74.6721	+72.1042
Ring	-214.0585	-214.6080	-215.3706
TSD	-75.3379	-52.9394	-64.1647
TSD (XMC)	-79.2685	-82.7942	-91.6418
TSD (SF)	-73.8945	-59.8000	-70.8585
vdW	-485.9308	-415.6403	-422.8311
$CH_2O + RNCO$	-467.1902	-400.6310	-410.8868
TSH	-87.2097	-86.4186	-89.6216
TSH (XMC)	-83.0993	-76.9544	-79.3906
TSH (SF)	-77.2400	-83.6488	-85.2482
CH(O)NHC(O)R	-549.1265	-547.6458	-551.8390
TSCO	-370.0134	-372.3841	
vdW	-503.5412	-496.2511	
ImH+CO	-482.5700	-476.9735	
TSH_2	-202.5222	-200.6007	
RC(O)NCO + H_2	-462.6770	-464.2032	

Table S7: Product yields of the ME calculations for the XMC-DPT2, SF-TDDFT and CCSD(T) energies for $CH_2OO + HCN$.
D: 3(R)-1,2,4-Dioxazole. I: Isocyanate + Formaldehyde. Im: Imidic Acid + CO

HCN	XMC	(298 K)		XMC	(240 K)	
p (torr)	D	I	Im	D	I	Im
1	0.00	0.55	0.45	0.00	0.55	0.45
⋮						
200	0.00	0.55	0.45	0.00	0.54	0.46
300	0.00	0.54	0.46	0.00	0.54	0.46
⋮						
760	0.00	0.54	0.46	0.00	0.54	0.46
	SF	(298 K)		CCSD(T)	(298 K)	
p (torr)	D	I	Im	D	I	Im
1	0.00	0.55	0.45	0.00	0.34	0.66
⋮						
760	0.00	0.55	0.45	0.00	0.34	0.66

Table S8: Product yields of the ME calculations for the XMC-DPT2, SF-TDDFT and CCSD(T) energies for $CH_2OO + CH_3CN$.
D: 3(R)-1,2,4-Dioxazole. I: Isocyanate + Formaldehyde. Im: Imidic Acid + CO

CH_3CN	XMC	(298 K)		XMC	(240 K)	
p (torr)	D	I	Im	D	I	Im
1	0.00	0.62	0.38	0.00	0.62	0.38
⋮						
200	0.01	0.62	0.37	0.03	0.60	0.37
300	0.02	0.61	0.37	0.06	0.58	0.35
400	0.04	0.60	0.36	0.10	0.56	0.34
600	0.08	0.58	0.35	0.18	0.51	0.31
760	0.11	0.56	0.33	0.24	0.47	0.28
	SF	(298 K)		CCSD(T)	(298 K)	
p (torr)	D	I	Im	D	I	Im
1	0.00	0.03	0.97	0.00	0.01	0.99
⋮						
200	0.01	0.03	0.96	0.00	0.01	0.99
300	0.02	0.03	0.95	0.01	0.01	0.99
400	0.04	0.02	0.94	0.02	0.01	0.98
600	0.08	0.02	0.90	0.04	0.01	0.95
760	0.11	0.02	0.86	0.06	0.01	0.93

Table S9: product yields of the ME calculations for the XMC-DPT2, SF-TDDFT and CCSD(T) energies for $CH_2OO + C_2H_5CN$.
D: 3(R)-1,2,4-Dioxazole. I: Isocyanate + Formaldehyde. Im: Imidic Acid + CO

C_2H_5CN	XMC	(298 K)		XMC	(240 K)	
p (torr)	D	I	Im	D	I	Im
1	0.00	0.93	0.07	0.00	0.93	0.07
40	0.19	0.75	0.05	0.37	0.59	0.04
100	0.43	0.53	0.04	0.64	0.34	0.02
200	0.62	0.35	0.02	0.79	0.20	0.01
300	0.71	0.27	0.02	0.85	0.14	0.01
400	0.77	0.21	0.01	0.88	0.11	0.01
600	0.84	0.15	0.01	0.92	0.07	0.00
760	0.87	0.13	0.01	0.94	0.06	0.00
	SF	(298 K)		CCSD(T)	(298 K)	
p (torr)	D	I	Im	D	I	Im
1	0.01	0.19	0.80	0.00	0.07	0.93
40	0.65	0.06	0.29	0.37	0.02	0.46
100	0.83	0.03	0.14	0.74	0.01	0.25
200	0.90	0.02	0.08	0.85	0.01	0.14
300	0.93	0.01	0.05	0.90	0.00	0.10
400	0.95	0.01	0.04	0.92	0.00	0.07
600	0.97	0.01	0.03	0.95	0.00	0.05
760	0.97	0.00	0.02	0.96	0.00	0.04

Criegee + Nitrile Association Rate

In Elsamra *et.al.*'s computational study on the reaction between CH_2OO and simple carbonyls, the high-pressure capture rate of the two molecules calculated using Long-Range Transition State Theory was found to have effectively no impact on the overall reaction kinetics at pressures from 1 to 10^{10} torr around $T = 298$ K, the outer capture rate being three orders of magnitude faster than the total reaction rate.[S12] It was thus estimated that the association of CH_2OO and RCN behaves similarly enough to $CH_2OO + R_1R_2C{=}O$ association that we may use Elsamra *et al.*'s capture rate equation of $k_{outer}^{p\to\infty}(T) = (7.17 + 21.39T^{-\frac{1}{6}} + 3.3T^{\frac{1}{6}}) \cdot 10^{-10}$ cm^3 molecule^{-1} s^{-1}, for the $CH_2OO + CH_3CHO$ association, which at our experimental temperature range (233-360 K) stays approximately constant at $k_{outer}^{p\to\infty} \approx 2.4 \cdot 10^{-9}$ cm^3 molecule^{-1} s^{-1} with less than 0.1 % variability. Here it is

worth pointing out that there is a possible misprint in the article, as the dipole-induced dipole interaction term in $k_{outer}^{p\to\infty}(T)$ should also be proportional to $T^{\frac{1}{6}}$. If we assume the equation should read $7.17T^{\frac{1}{6}}$ rather than 7.17, then the total capture rate approximates to $k_{outer}^{p\to\infty} \approx 3.5 \cdot 10^{-9}$ cm^3 molecule^{-1} s^{-1} with a 4 % variability within the experimental temperature range. Nevertheless, as this value neither impacts the overall rate of the reaction nor the initial energy distribution of the product, more accurate CH_2OO + RCN capture rates were not calculated.

Calculation of Lennard-Jones parameters

The Lennard-Jones parameters for the relevant compounds (The van der Waals complex RC, the dioxazole and the formyl formamide product) were determined using the same method as detailed by Cao *et. al.*[S13] The parameters are determined from the critical temperature (T_c), critical pressure (p_c), and boiling point (T_b). which are calculated using the Joback group additivity model[S14] (σ in Å. ϵ in the same energy unit as kT):

$$\sigma = 2.44\left(\frac{\mathrm{J}}{\mathrm{K}}\right)^{\frac{1}{3}}\left(\frac{T_c}{p_c}\right)^{\frac{1}{3}} \qquad \epsilon = 0.77kT_c \tag{2}$$

This method was modified slightly for the reactive complexes, due to the availability of experimental data for the individual molecules. Joback's model does not account for interactions between the functional groups in a molecule. As such, the discrepancy between the experimental quantities T_c and p_c and the Joback modelled parameters were fully attributed to these interactions, and 'solved for' by adding an extra additive term to Joback's equations (3). The reactant complexes were thus treated as single molecules including the functional groups and 'interaction terms' of both molecules. The equations with which the complex's

thermodynamic quantities are calculated are thus:

$$T_c = \frac{T_b}{0.584 + 0.965\left(\sum_i T_i + T_A + T_B\right) - \left(\sum_i T_i + T_A + T_B\right)^2} \tag{3a}$$

$$p_c = \frac{1}{\left(0.113 + 0.0032N - \left(\sum_i p_i + p_A + p_B\right)\right)^2} \tag{3b}$$

$$T_b = 198 + \left(\sum_i T_{bi} + T_{bA} + T_{bB}\right) \tag{3c}$$

The temperature unit is Kelvin and the pressure unit is bar in these equations. $\sum_i X_i$ are sums of Joback's group contributions, whereas X_A and X_B are correction terms added to the equations such that they accurately predict the experimental values of T_c and p_c for individual molecules. This trick still leaves the intermolecular contributions of specific groups unaccounted for, but the method was deemed slightly more accurate than simply relying on Joback group additivity. The used LJ parameters along with all intermediary steps are listed in Table S10.

Table S10: Calculated thermodynamic quantities and derived Lennard-Jones parameters of the relevant compounds. F.Am. is short for Formyl amide. vdW1 refers to the $CH_2OO + RCN$ van der Waals complex, whereas vdW2 refers to the CO + Iminic Acid van der Waals complex.

Nitrile	Species	N	T_b (K)	T_c (K)	p_c (bar)	$\frac{\epsilon}{k_B}$ (K)	σ (Å)
HCN	vdW1	8	377.27	633.46	49.76	487.8	5.70
HCN	Ring	8	372.07	593.85	72.06	457.3	4.93
HCN	F.Am.	8	392.85	582.41	64.10	456.3	4.88
HCN	vdW2	8	282.55	543.02	38.97	418.1	5.87
CH_3CN	vdW1	11	438.27	650.08	290.61	500.6	3.19
CH_3CN	Ring	11	398.93	619.70	60.19	477.2	5.31
CH_3CN	F.Am.	11	420.94	614.48	54.39	488.9	5.20
CH_3CN	vdW2	11	305.31	603.38	44.0	464.6	5.84
C_2H_5CN	vdW1	14	456.62	664.57	388.10	511.7	2.92
C_2H_5CN	Ring	14	421.81	639.96	52.13	492.8	5.63
C_2H_5CN	F.Am.	14	443.82	635.48	47.43	499.3	5.55

Sensitivity Analysis of Energy transfer model

As covered in the main text, the model used in the ME model was exponential down energy transfer with Lennard-Jones potential. This model requires three more or less empirical parameters: The Lennard-Jones σ and ϵ values, as well as the average transferred energy per collision, $\langle \Delta E \rangle_{\mathrm{down}}$. The determination of the LJ parameters we have already covered, but for $\langle \Delta E \rangle_{\mathrm{down}}$ an estimation will have to do. In the supplementary material of Jasper *et.al.*,[S15] $\langle \Delta E \rangle_{\mathrm{down}}$ values computed using Molecular Dynamics are found for various alkanes for several bath gases at temperatures 300 K and 1000 K. Out of the compounds covered in the article, the one most resembling 1,2,4-dioxazole is cyclopentane, for which the values $\langle \Delta E \rangle_{\mathrm{down}} = 242$ cm^{-1} and $\langle \Delta E \rangle_{\mathrm{down}} = 298$ cm^{-1} are listed for Ar and O_2 bath gas specifically. From these values, $\langle \Delta E \rangle_{\mathrm{down}} \approx 260$ cm^{-1} can be inferred for N_2 gas using conversion factors presented in the main text. Substituted ring structures were not covered at all in Jasper *et.al.*'s article, thus values for the larger two systems were inferred by comparing the $\langle \Delta E \rangle_{\mathrm{down}}$ differences values between pentene, hexene and heptene and adding that number to 1,2,4-dioxazole's parameter. Energy transfer parameters were needed for the product formyl formamides as well, as the decomposition reaction resulting in them is reversible. For these, the values for pentene, hexene and heptene were simply used. The parameters for all compounds with unimolecular reactions in the ME model are presented in Table S11

Table S11: The $\langle \Delta E \rangle_{\mathrm{down}}$ parameter used for each compound with unimolecular reactions in the ME model. The unit is cm^{-1}. vdW1 and vdW2 have the same meaning as in table S10.

R	vdW1	Ring	CH(O)NHC(O)R	vdW2
HCN	260	260	490	490
CH_3CN	310	310	540	540
C_2H_5CN	340	340	570	

To stress-test the validity of this approximation, a sensitivity analysis of the dioxazole $\langle \Delta E \rangle_{\mathrm{down}}$ was performed, at values of ± 50 cm^{-1} and $\pm$ 100cm^{-1}. As seen in Table S12, varying the parameter does not change the results qualitatively.

Table S12: Sensitivity of the dioxazole product yield to changes in the energy transfer parameter $\langle \Delta E \rangle_{\text{down}}$. The results are calculated using the E_{XMC} potential surface with 760 torr pressure and 298.15 K temperature.

R	-100 cm^{-1}	-50 cm^{-1}	$+50$ cm^{-1}	$+100$ cm^{-1}
HCN	$5.2 \cdot 10^{-6}$	$8.2 \cdot 10^{-5}$	$1.4 \cdot 10^{-3}$	$3.0 \cdot 10^{-3}$
CH_3CN	0.08	0.13	0.23	0.27
C_2H_5CN	0.81	0.85	0.89	0.90

Comparison of RRKM and ILT results

As mentioned in the Computational Methods section of the main article, the association rate of CH_2OO and RCN into a van der Waals complex is set to a constant value of $2.36 \cdot 10^{-9}$ s^{-1} and the subsequent ring closure is modelled using RRKM theory.[S16] This approach builds on a couple of physical assumptions which may not be accurate. Firstly, it neglects the possibility of a long-range centrifugal barrier.[S17] Secondly, it assumes the van der Waals complex's energy distribution is uniform enough to be modelled in the microcanonical ensemble. Thirdly, it assumes that the energy saddle point for the ring closure (TS1) found using DFT is also the saddle point in Gibbs free energy, which might not be the case, as ring closure is an entropy-controlled process. For these reasons, two additional ME simulations were performed for the $CH_2OO + CH_3CN$ and $CH_2OO + C_2H_5CN$ reactions, in which the association and ring closure are modelled together using the Inverse Laplace Transform method[S18] where the ILT parameters are determined by a least-squares fit of the experimental data. The ILT results are presented in Table S13 and the corresponding results from the main article are also presented here, in Table S14. As seen from the tables, both methods result in similar product yields. The only noticeable difference is that the ILT simulations procude a mildly less stable $C_4H_7O_2N$ dioxazole. This was taken as indication that the compromises made in the ME model do not change our conclusions.

Table S13: Product yields of the ME calculations for the XMC-QDPT2 potential surface using the ILT method. I: Isocyanate + Formaldehyde. Im: Imidic acid.

XMC		CH_3CN			C_2H_5CN		
p (torr)	T (K)	D	I	Im	D	I	Im
10	233	0.00	0.61	0.39	0.02	0.92	0.07
10	253	0.00	0.61	0.39	0.01	0.92	0.07
10	273	0.00	0.61	0.39	0.01	0.92	0.07
10	296	0.00	0.61	0.39	0.01	0.92	0.07
10	320	0.00	0.61	0.39	0.01	0.92	0.07
10	360	0.00	0.61	0.39	0.00	0.93	0.07
50	296	0.00	0.61	0.39	0.16	0.79	0.06
100	296	0.00	0.61	0.39	0.33	0.62	0.05
200	296	0.00	0.61	0.38	0.53	0.44	0.03

Table S14: Product yields of the ME calculations for the XMC-QDPT2, SF-TDDFT and CCSD(T) energies.
D: 3(R)-1,2,4-Dioxazole. I: Isocyanate + Formaldehyde. Im: Imidic acid.

XMC		CH_3CN			C_2H_5CN		
p (torr)	T (K)	D	I	Im	D	I	Im
40	298.15	0.00	0.62	0.38	0.19	0.75	0.05
100	298.15	0.00	0.62	0.38	0.43	0.53	0.04
200	298.15	0.01	0.62	0.37	0.62	0.35	0.02

References

(S1) Roehl, C. M.; Burkholder, J. B.; Moortgat, G. K.; Ravishankara, A.; Crutzen, P. J. Temperature dependence of UV absorption cross sections and atmospheric implications of several alkyl iodides. *J. GeoPhys. Res.* **1997**, *102*, 12819–12829.

(S2) Rattigan, O. V.; Shallcross, D. E.; Anthony Cox, R. UV absorption cross-sections and atmospheric photolysis rates of CF_3I, CH_3I, C_2H_5I and CH_2ICl. *J. Chem. Soc., Faraday Trans.* **1997**, *93*, 2839–2846.

(S3) Mössinger, J.; Shallcross, D.; Cox, R. UV-visible absorption cross-section and atmospheric lifetimes of CH_2Br_2, CH_2I_2 and CH_2BrI. *J. Chem. Soc. Faraday Trans.* **1998**, *94*, 1391–1396.

(S4) Renner, T. A.; Blander, M. A study of dimerization in acetonitrile vapor by measurement of thermal conductivity. *J. Phys. Chem.* **1977**, *81*, 857–861.

(S5) Legon, A.; Millen, D.; Mjöberg, P. The hydrogen cyanide dimer: identification and structure from microwave spectroscopy. *Chem. Phys. Lett.* **1977**, *47*, 589–591.

(S6) Frisch, M. J.; Trucks, G. W.; Schlegel, H. B.; Scuseria, G. E.; Robb, M. A.; Cheeseman, J. R.; Scalmani, G.; Barone, V.; Petersson, G. A.; Nakatsuji, H.; Li, X.; Caricato, M.; Marenich, A. V.; Bloino, J.; Janesko, B. G.; Gomperts, R.; Mennucci, B.; Hratchian, H. P.; Ortiz, J. V.; Izmaylov, A. F.; Sonnenberg, J. L.; Williams-Young, D.; Ding, F.; Lipparini, F.; Egidi, F.; Goings, J.; Peng, B.; Petrone, A.; Henderson, T.; Ranasinghe, D.; Zakrzewski, V. G.; Gao, J.; Rega, N.; Zheng, G.; Liang, W.; Hada, M.; Ehara, M.; Toyota, K.; Fukuda, R.; Hasegawa, J.; Ishida, M.; Nakajima, T.; Honda, Y.; Kitao, O.; Nakai, H.; Vreven, T.; Throssell, K.; Montgomery, J. A., Jr.; Peralta, J. E.; Ogliaro, F.; Bearpark, M. J.; Heyd, J. J.; Brothers, E. N.; Kudin, K. N.; Staroverov, V. N.; Keith, T. A.; Kobayashi, R.; Normand, J.; Raghavachari, K.; Rendell, A. P.; Burant, J. C.; Iyengar, S. S.; Tomasi, J.; Cossi, M.; Millam, J. M.; Klene, M.; Adamo, C.; Cammi, R.; Ochterski, J. W.; Martin, R. L.; Morokuma, K.; Farkas, O.; Foresman, J. B.; Fox, D. J. Gaussian˜16 Revision C.01. 2016; Gaussian Inc. Wallingford CT.

(S7) Granovsky, A. A. Firefly version 8.0.0. `http://classic.chem.msu.su/gran/firefly/index.html`.

(S8) Barca, G. M. J.; Bertoni, C.; Carrington, L.; Datta, D.; De Silva, N.; Deustua, J. E.; Fedorov, D. G.; Gour, J. R.; Gunina, A. O.; Guidez, E.; Harville, T.; Irle, S.; Ivanic, J.; Kowalski, K.; Leang, S. S.; Li, H.; Li, W.; Lutz, J. J.; Magoulas, I.; Mato, J.; Mironov, V.; Nakata, H.; Pham, B. Q.; Piecuch, P.; Poole, D.; Pruitt, S. R.; Rendell, A. P.; Roskop, L. B.; Ruedenberg, K.; Sattasathuchana, T.; Schmidt, M. W.; Shen, J.; Slipchenko, L.; Sosonkina, M.; Sundriyal, V.; Tiwari, A.; Galvez Vallejo, J. L.;

Westheimer, B.; Wloch, M.; Xu, P.; Zahariev, F.; Gordon, M. S. Recent developments in the general atomic and molecular electronic structure system. *J. Chem. Phys.* **2020**, *152*, 154102.

(S9) Ayala, P. Y.; Schlegel, H. B. Identification and treatment of internal rotation in normal mode vibrational analysis. *J. Chem. Phys.* **1998**, *108*, 2314–2325.

(S10) Granovsky, A. A. Extended multi-configuration quasi-degenerate perturbation theory: The new approach to multi-state multi-reference perturbation theory. *J. Chem. Phys.* **2011**, *134*, 214113.

(S11) Shao, Y.; Head-Gordon, M.; Krylov, A. I. The spin–flip approach within time-dependent density functional theory: Theory and applications to diradicals. *J. Chem. Phys.* **2003**, *118*, 4807–4818.

(S12) Elsamra, R. M. I.; Jalan, A.; Buras, Z. J.; Middaugh, J. E.; Green, W. H. Temperature- and Pressure-Dependent Kinetics of $CH_2OO + CH_3COCH_3$ and $CH_2OO + CH_3CHO$: Direct Measurements and Theoretical Analysis. *Int. J. Chem. Kinet.* **2016**, *48*, 474–488.

(S13) Gao, C. W.; Allen, J. W.; Green, W. H.; West, R. H. Reaction Mechanism Generator: Automatic construction of chemical kinetic mechanisms. *Computer Physics Communications* **2016**, *203*, 212–225.

(S14) Joback, K.; Reid, R. Estimation of Pure-component Properties From Group-Contributions. *Chemical Engineering Communications* **1987**, *57*, 233–243.

(S15) Jasper, A. W.; Oana, C. M.; Miller, J. A. "Third-Body" collision efficiencies for combustion modeling: Hydrocarbons in atomic and diatomic baths. *Proc. Combust. Inst.* **2015**, *35*, 197–204.

(S16) Marcus, R. A. Unimolecular Dissociations and Free Radical Recombination Reactions. *J. Chem. Phys.* **1952**, *20*, 359–364.

(S17) Georgievskii, Y.; Klippenstein, S. J. Long-range transition state theory. *J. Chem. Phys.* **2005**, *122*, 194103.

(S18) Venkatesh, P. K.; Carr, R. W.; Cohen, M. H.; Dean, A. M. Microcanonical Transition State Theory Rate Coefficients from Thermal Rate Constants via Inverse Laplace Transformation. *J. Phys. Chem. A* **1998**, *102*, 8104–8115.
