# Supplementary material for: An Experimental and Master Equation Investigation of Kinetics of the CH2OO + RCN Reactions (R = H, CH3, C2H5) and Their Atmospheric Relevance
Source: J Phys Chem A. 2023 Jan 5;127(2):477–88. doi: 10.1021/acs.jpca.2c07073 (PMC9869398; doi:10.1021/acs.jpca.2c07073)
Supplement: Supplementary file 2 — jp2c07073_si_002.zip [file jp2c07073_si_002.zip › Readme.pdf]

Supporting Information: An Experimental and Master-Equation Investigation of Kinetics of the CH_2OO + RCN Reactions (R = H, CH_3, C_2H_5) and their Atmospheric Relevance

Lauri Franzon[a], Jari Peltola[a], Rashid Valiev, Niko Vuorio, Theo Kurten, and Arkke Eskola*

Department of Chemistry, University of Helsinki,

P.O. Box 55 (A.I. Virtasen aukio 1), 00014 Helsinki, Finland

E-mail: arkke.eskola@helsinki.fi

[0a] *These authors contributed equally to this work*

Table of Contents

A description of the contents of each directory:

- **CCSD(T)** Output files of the CCSD(T)-F12a/VDZ-F12 single point energy calculations for all chemical species on the potential energy surface.
- **MESMER** Master Equation input and output files.
  - **DeltaEDownSensitivity** The ME simulations related whose results are presented in Table S12.
  - **ILT-ExpFit** The ME simulations whose results are presented in Table S13.
  - **ProductDistribution** The ME simulations whose results are presented in Table S7-S9.
  - **RateComparison** The ME simulations whose results are presented in Table 6.
- **wb97XD** Output files of the ωB97X-D/aug-cc-pVTZ quantum chemistry calculations.
  - **HinderedRotor** Hindered rotor scans for all chemical species included in the Master Equation with $-CH_3$ and $-C_2H_5$ functionalities. The results are presented in Table S3.
  - **IRC** IRC reaction path calculations for the CH_2OO + RCN ring closure and 1,2,4-(R)-dioxazole decomposition/rearrangement reactions.
  - **Optimization+Freq** Geometry optimization and frequency analysis for all species on the potential energy surface. The results are presented in Table S6.
